# Developing a Yeast Platform Strain for an Enhanced Taxadiene Biosynthesis by CRISPR/Cas9

**DOI:** 10.3390/metabo11030147

**Published:** 2021-03-03

**Authors:** Joseph C. Utomo, Fabio C. Chaves, Philippe Bauchart, Vincent J. J. Martin, Dae-Kyun Ro

**Affiliations:** 1 Department of Biological Science, University of Calgary, Calgary, AB T2N1N4, Canada; joseph.utomo1@ucalgary.ca; 2 Programa de Pós-graduação em Ciência e Tecnologia de Alimentos, Departamento de Ciência e Tecnologia Agroindustrial, Faculdade de Agronomia Eliseu Maciel, Universidade Federal de Pelotas, Caixa Postal 354, Pelotas CEP 96010-900, Brazil; fabio.chaves@ufpel.edu.br; 3 Department of Biology, Centre for Applied Synthetic Biology, Concordia University, Montréal, QC H4B1R6, Canada; pbauchart@lallemand.com (P.B.); vincent.martin@concordia.ca (V.J.J.M.)

**Keywords:** paclitaxel, taxadiene, *S*. *cerevisiae*, metabolic engineering, diterpenoids

## Abstract

Paclitaxel is an important diterpenoid commonly used as an anticancer drug. Although the paclitaxel biosynthetic pathway has been mostly revealed, some steps remain to be elucidated. The difficulties in plant transformations and the scarcity of the precursor of paclitaxel, (+)-taxa-4(5), 11(12)-diene (taxadiene), have hindered the full comprehension of paclitaxel biochemistry and, therefore, its production by biotechnological approaches. One solution is to use the budding yeast, *Saccharomyces cerevisiae*, as a platform to elucidate the paclitaxel biosynthesis. As taxadiene is a diterpenoid, its common precursor, geranylgeranyl pyrophosphate (*GGPP*), needs to be increased in yeast. In this study, we screened various *GGPP* synthases (*GGPPS*) to find the most suitable *GGPPS* for taxadiene production in yeast. We also optimized the taxadiene production by increasing the flux toward the terpenoid pathway. Finally, to remove selection markers, we integrated the required genes using a CRISPR/Cas9 system in the yeast genome. Our result showed that a titer of 2.02 ± 0.40 mg/L (plasmid) and 0.41 ± 0.06 mg/L (integrated) can be achieved using these strategies. This platform strain can be used to readily test the gene candidates for microbial paclitaxel biosynthesis in the future.

## 1. Introduction

Global cancer cases are increasing with an estimation of more than 18 million cases and almost 10 million deaths in 2018 [1]. The ever-increasing cases of cancer necessitate a reliable and inexpensive supply of anticancer drugs. One of the most prescribed anticancer drugs is paclitaxel or commonly known as its commercial brand, Taxol. It acts by stabilizing tubulin and inducing cytokines that cause mitosis prevention and cell growth inhibition in cancer cells [2,3,4]. Due to its selective and potent mechanism of action, paclitaxel-based drugs have been used in chemotherapy to treat various cancers, including breast, lung, ovarian, and sarcoma [2,5]. This potent compound is naturally produced in the bark of the Pacific yew tree in minute quantities [3]. Commercially, there are two approaches to achieve the supply of paclitaxel, chemical (semi-) synthesis [6,7] and plant cell cultivation, of which the latter is more popular due to the economic viability of cell culture [8]. Despite these commercial advances, the tools to improve paclitaxel bio-production are limited. The major hurdles limiting the improvement of paclitaxel yield are the unreliability of *Taxus* spp. transformation [9] and incomplete understandings of paclitaxel biosynthesis at the molecular level. These limitations hamper the cost-effective supply of paclitaxel through a biotechnological means [10].

**Figure 1:**
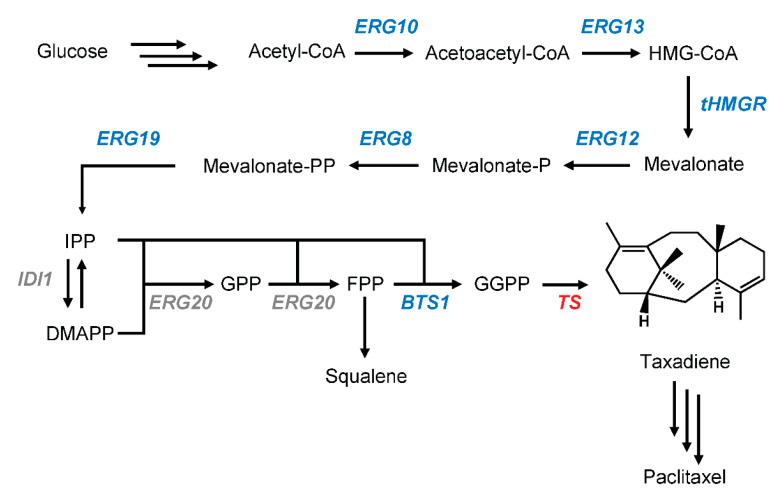
A schematic diagram of the taxadiene-producing yeast construction in *S*. *cerevisiae*. The overexpressed genes are shown in blue. The heterologous *taxadiene synthase* is shown in red. The endogenous genes without any modification are shown in grey.

Paclitaxel is classified as a diterpene, 20-carbon molecule derived from acetyl-CoA via the terpenoid pathway (Figure 1). As all diterpenoids, paclitaxel is synthesized from the universal diterpene precursor, geranylgeranyl diphosphate (*GGPP*), followed by decorations of the backbones by cytochromes P450 monooxygenases (P450s) and transferases [11]. The first step in paclitaxel biosynthesis is the conversion of *GGPP* into its diterpene backbone, (+)-taxa-4(5),11(12)-diene or taxadiene, by an enzyme called taxadiene synthase (TS) [12]. This step is followed by at least 18 enzymatic steps of P450s and transferases [11,12]. Most steps in the paclitaxel biosynthetic pathway have been described previously, the majority of which was brilliantly conducted by the Croteau lab. However, several steps remain missing and unclear [13]. For example, steps between taxadiene-5α,10β-diol 5-acetate and 2-debenzoyl taxane have not been determined, while the ones that had been proposed could not be placed accurately between these two intermediates [13,14,15]. A similar question can be raised for an enzyme called taxoid 13α-hydroxylase, which has been characterized but cannot be precisely placed in the pathway [16]. Additionally, recent studies based on heterologous expression data question the appointed first enzymatic step after taxadiene, originally discovered by the Croteau group, who characterized the enzyme involved in the first oxygenation of taxadiene [17,18,19]. To shed light on this divergence and to further elucidate the paclitaxel biosynthesis pathway, a more comprehensive study needs to be performed. Unfortunately, taxadiene is not available commercially and its synthesis is impractical. One solution for this issue is utilizing a microbial platform to study the paclitaxel biosynthetic pathway.

Several microbial platforms have been developed to produce taxadiene. Expectedly, the first attempt was in the prokaryote workhorse, *E*. *coli* [20,21]. Although the production of taxadiene in *E*. *coli* is promising, it is not an ideal system for studying the paclitaxel biosynthetic pathway because many steps in the paclitaxel biosynthetic pathway are catalyzed by P450s, which require endoplasmic reticulum anchoring, unavailable in prokaryotes [13,20,21]. Nonetheless, some attempts to follow the first step have been conducted in *E*. *coli* with a great success, producing more than 500 mg/L of taxane. This was achieved by swapping the N-terminus of P450 with an eight-residue peptide (8RP) from bovine as demonstrated by Biggs et al. [21,22]. Despite this success, the mandatory modifications of the heterologous P450s enzymes for expression in *E*. *coli* will add complications for studying the ambiguous paclitaxel biosynthetic pathway. Therefore, using the budding yeast (*Saccharomyces cerevisiae*) as a platform to investigate the paclitaxel biosynthetic pathway might be more reliable and advantageous over *E*. *coli*.

Production of taxadiene in *S*. *cerevisiae* has been previously attempted [23,24,25,26]. The first attempt was performed by the Croteau lab and successfully yielded 1 mg/L taxadiene. Currently, the highest reported titer of taxadiene production is ~57 mg/L, which was achieved by optimizing TS expression and providing multi-copies of *TS* in the genome [26]. Since *S*. *cerevisiae* does not synthesize diterpenoid-derived metabolites, one essential modification is enhancing the production of *GGPP* [27]. Previous studies utilized characterized geranylgeranyl diphosphate synthase (*GGPPS*) from *Taxus* spp., archaea, and carotenoid-producing bacteria (*Escherichia vulneris*) [24,25,26]. However, different *GGPPSs* have not been comparatively examined to enhance taxadiene production in yeast.

In this study, our goal was to build a taxadiene-producing yeast strain that can be used as a microbial platform to elucidate the remaining steps of the paclitaxel biosynthetic pathway. To achieve this, we screened various *GGPPS* overexpression impacts on taxadiene titer in yeast and integrated necessary genes to make an efficient taxadiene-producing yeast strain without plasmid transformations. By achieving this goal, the subsequent enzymes in the paclitaxel biosynthesis pathway can be studied with greater efficiency without involving selection markers for the yeast plasmids.

## 2. Results and Discussion

### 2.1. pIPP Construction to Increase the Flux towards Terpenoid Pathway

**Figure 2:**
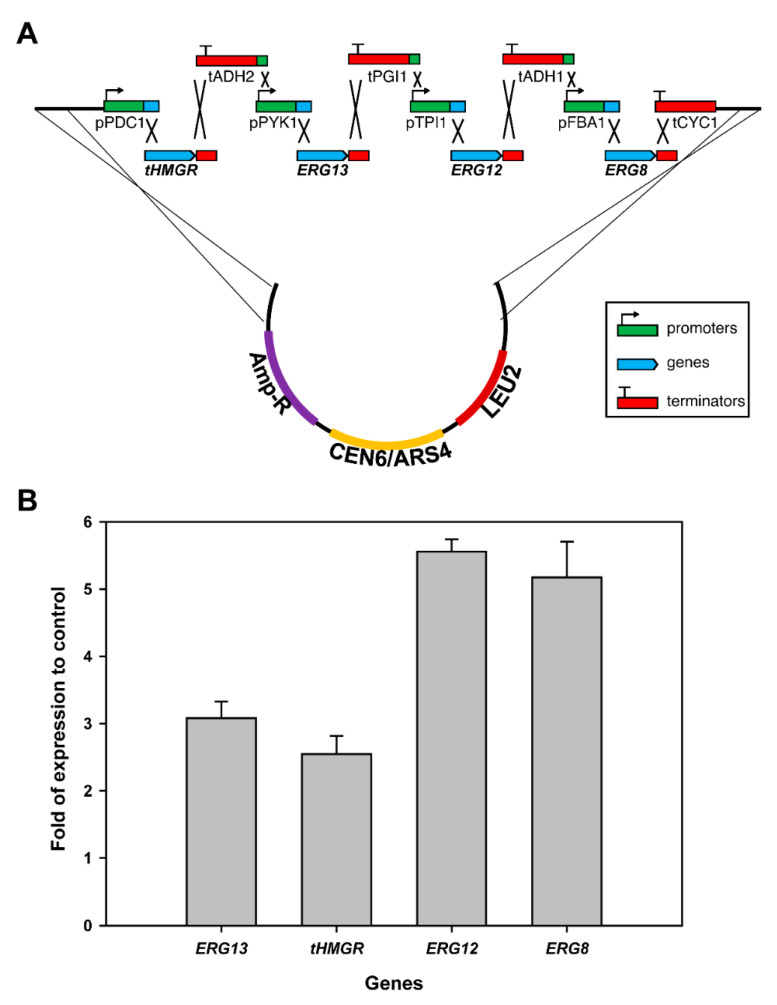
(**A**) A schematic diagram of the in vivo construction of pIPP plasmid encoding four middle genes in the MVA pathway. Abbreviations: **p**: promoter; **t**: terminator; PDC: pyruvate decarboxylase; ADH: alcohol dehydrogenase; PYK: pyruvate kinase; PGI: phosphoglucose isomerase; TPI: triose phosphate isomerise; FBA: fructose 1,6-bisphosphate aldolase; CYC: cytochrome c; tHMGR: truncated 3-hydroxy-3-methylglutaryl-CoA reductase. (**B**) The transcript abundance of the four genes were measured in both non-transgenic yeast (control) and transgenic yeast by quantitative PCR. Fold increases in each transcript in transgenic yeast, relative to those from control yeast, were calculated after the transcript levels from the control yeast were set to one. In all transcripts, differences of gene expression in transgenic and control yeasts were statistically significant (*p*-value < 0.01).

To build a taxadiene-producing yeast strain, the endogenous yeast mevalonate (MVA) pathway needs to be enhanced (Figure 1). It has been shown that the truncated and soluble version of 3-hydroxy-3-methylglutaryl-CoA reductase (tHMGR) can improve the production of terpenoid in yeast as it is a rate-limiting and a key regulatory enzyme in the MVA pathway [28,29]. However, the overexpression of one rate-limiting enzyme might not be sufficient for the overall flux increase in the MVA pathway as shown in the glycolytic pathway [30,31]. To increase the overall flux toward the terpenoid pathway, we decided to first overexpress the four-middle genes in the MVA pathway (*ERG13*, *tHMGR*, *ERG12*, and *ERG8*) using a plasmid. We utilized the DNA assembler method [32] to construct the pIPP plasmid that contains these genes under constitutive promoters with a CEN6/ARS4 origin of replication (Figure 2). To test the expression of these genes in yeast, the quantitative polymerase chain reaction (qPCR) was performed. The transcript abundance of these genes in transgenic yeast, relative to those in wild-type yeast, showed that the transformation of these genes into yeast indeed increased their transcript levels ranging from 2- to 5-fold (Figure 2). This result indicated that pIPP can be used to increase flux towards taxadiene production.

### 2.2. Various GGPPS Overexpression

Yeast produces only a small amount of *GGPP*, which is endogenously used for protein prenylations, while no secondary diterpene metabolites are produced in yeast [27]. Therefore, overexpression of a *GGPPS* is essential to enhance *GGPP* (C_20_) production. Generally, there are two types of substrate specificity in different *GGPPSs*. Some *GGPPSs* have a higher affinity towards one FPP (C_15_) and one IPP (C_5_) utilization to synthesize *GGPP*, whereas others have a higher affinity towards one DMAPP (C_5_) and three IPPs (C_5_) to synthesize *GGPP* [33]. To get a clear conclusion on the most suitable *GGPPS* for the production of taxadiene in yeast, we screened *GGPPS* from various sources: yeast (*BTS1*), algae (*CrGGPPS*), pepper (*CaGGPPS*), modified yeast FPP synthase (*mERG20*) [34], and archaea (*SaGGPPS*). Two of those (*mERG20* and *SaGGPPS*) were used to increase diterpenoid production in yeast, but their impacts on diterpene production relative to each other have not been reported [24,34], while the others have not been studied for diterpene production in yeast. These genes were codon-optimized for yeast and overexpressed simultaneously with *TS* in the same plasmid, together with pIPP, to assess the effect of these genes on taxadiene production. Expression of all *GGPPSs* was checked by immunoblot analysis (Figure S1).

Most of the previous studies on improving taxadiene production in yeast did not use *BTS1* overexpression since *BTS1*, like human *GGPPS*, is active only when FPP is present and may compete with squalene synthase for the common substrate, FPP [24,25,26,27]. However, we had overexpressed *BTS1* to enhance diterpenes production in yeast and a significant increase in diterpene production was observed [35]. Therefore, we included *BTS1* to evaluate its impacts on taxadiene production in comparison to other *GGPPSs*. The second *GGPPS* is from thermoacidophilic archaea, *Sulfolobus acidocaldarius* (*SaGGPPS*). This *GGPPS* has been previously used to improve taxadiene production in yeast [24]. Different from *BTS1*, it shows a higher activity when utilizing DMAPP, although some activity is still observed when FPP is present [36]. The third *GGPPS* is from pepper, *Capsicum annum* (*CaGGPPS*). There is no kinetic velocity data for *CaGGPPS*, but it has been shown that *CaGGPPS* has a slightly higher affinity towards DMAPP compared to FPP [37]. Meanwhile, algae (*Chlamydomonas reinhardtii*) *GGPPS* activity has not been tested since its discovery, which was identified solely based on comparative genomics in the studies of carotenoid biosynthetic genes in *C*. *reinhardtii* [38]. To the best of our knowledge, this is the first study to examine *CrGGPPS* functionality to increase diterpene production in yeast. Finally, the yeast FPPS (*ERG20*) with a single mutation (*mERG20*) was included as it had been previously shown to improve *GGPP* production of diterpenoids in yeast [34].

**Figure 3:**
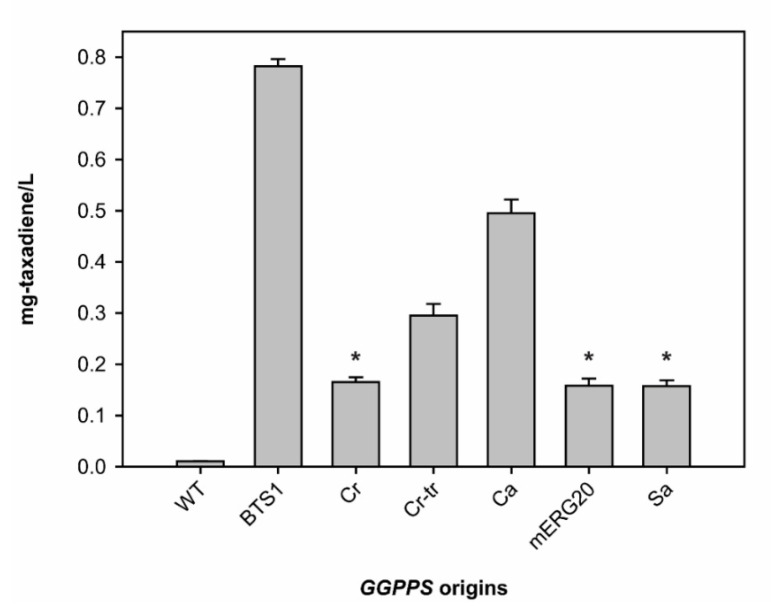
Taxadiene production titer using *S*. *cerevisiae* transformed with Tm-TS and pIPP with overexpression of different geranylgeranyl pyrophosphate synthases (*GGPPSs*) from various species. The asterisks indicate no significant differences between the data (*p* value > 0.05). The bars without asterisk have significant different between them (*p* value < 0.01). Abbreviations: WT, *Saccharomyces cerevisiae* strain BY4742 without overexpression of *GGPPS*; BTS1, *Saccharomyces cerevisiae GGPPS*; Cr, *Chlamydomonas reinhardtii GGPPS*; Cr-tr, truncated Cr; Ca, *Capsicum annuum GGPPS*; mERG20, mutant of yeast *FPPS*; Sa, *Sulfolobus acidocaldarius GGPPS*.

Here, we compared the effects of various *GGPPS* expression toward the production of taxadiene when they were transformed simultaneously with *TS* and pIPP. Our results showed that overexpression of endogenous yeast *GGPPS* (*BTS1*) led to the highest taxadiene production with 782 ± 14 μg/L, almost an 80-fold increase compared to the yeast without *GGPPS* overexpression (Figure 3). *BTS1* overexpression also showed a four-fold increase in taxadiene titer, compared to the yeast overexpressing *SaGGPPS*, which was used in a previous study [24]. Taxadiene production using the *BTS1* overexpression was also 80-fold higher than using *mERG20*. These data are contradictory to the report that showed overexpression of *mERG20* in yeast resulted in an over 80-fold increase in diterpene production compared to the *BTS1* overexpression [34]. The difference between our study and the previous one regarding BTS1 and mERG20 impacts on taxadiene titer could be due to a difference in the expression strategy. In the Ignea et al. study, they used a constitutive promoter for the diterpene synthase and a galactose inducible promoter for *GGPPS* in two different plasmids [34]. Meanwhile, we used the galactose inducible promoters for both *TS* and *GGPPS* in a single plasmid (pESC). The expression of both genes in a single plasmid, as we employed in this study, should lead to a more synchronized gene expression of diterpene synthase and *GGPPS*. Thus, additional factors such as differences in plasmid copy numbers can be eliminated, and the taxadiene titer differences between BTS1 and mERG20 overexpression are caused by their internal characteristics.

Another intriguing result is from the *C*. *reinhardtii GGPPS* (*CrGGPPS*). *C*. *reinhardtii* is a model organism to study microalgae and photosynthesis [38]. However, its *GGPPS* activity has not been evaluated. There is an indication from the sequence alignment with cyanobacteria *GGPPS* that *C*. *reinhardtii* may encode a preprotein (*CrGGPPS*), and a mature, truncated version of *GGPPS* (*Cr-trGGPPS*) is produced from the preprotein [38]. Thus, both versions were tested to examine their effects on taxadiene production in yeast. Figure 3 shows that indeed overexpression of the mature version (*Cr-trGGPPS*) resulted in a higher level of taxadiene production by almost 80% compared to the preprotein version (*CrGGPPS*). This result strongly suggested that *C*. *reinhardtii GGPPS* has a transit peptide that enables plastid-targeting, similar to other *GGPPS* in terrestrial plants [38], but its preprotein version still retains *GGPPS* activity. It is interesting to note that overexpression of the preprotein version of *CrGGPPS* resulted in a similar level of taxadiene production with both *SaGGPPS* and *mERG20* in yeast.

The second highest taxadiene production was achieved when *CaGGPPS* was overexpressed (Figure 3). *CaGGPPS* is one of the first plant *GGPPS* that was purified and identified while investigating carotenoid biosynthesis [37,39]. It is localized in pepper plastids and has a transit peptide [39]. We synthesized and overexpressed the mature form of *CaGGPPS*. The difference between *CaGGPPS* and BTS1 impacts on taxadiene production may be explained by the differences in substrate specificity of these two enzymes. While *CaGGPPS* is more promiscuous with a slight preference towards DMAPP [37], BTS1 strictly prefers FPP as the substrate [27]. Due to the strong activity exerted by yeast FPPS (ERG20), more FPP is available in yeast than DMAPP [40]. Hence, the BTS1 can readily use FPP, while DMAPP availability could be limited for *CaGGPPS* and other *GGPPS* that prefer DMAPP as a priming molecule. As different *GGPPSs* have different substrate preference, BTS1 might show the highest activity in yeast due to availability of the priming substrate (FPP), rather than its superior catalytic activity over other *GGPPSs* which use DMAPP and IPP as substrates. We presume that true activities of other *GGPPSs* need to be re-evaluated in the yeast engineered to produce abundant DMAPP to draw a fair conclusion regarding their catalytic activities. Nonetheless, our results here demonstrated that native BTS1 performs best with respect to in vivo production of taxadiene in yeast.

### 2.3. Optimization of Taxadiene Production

**Figure 4:**
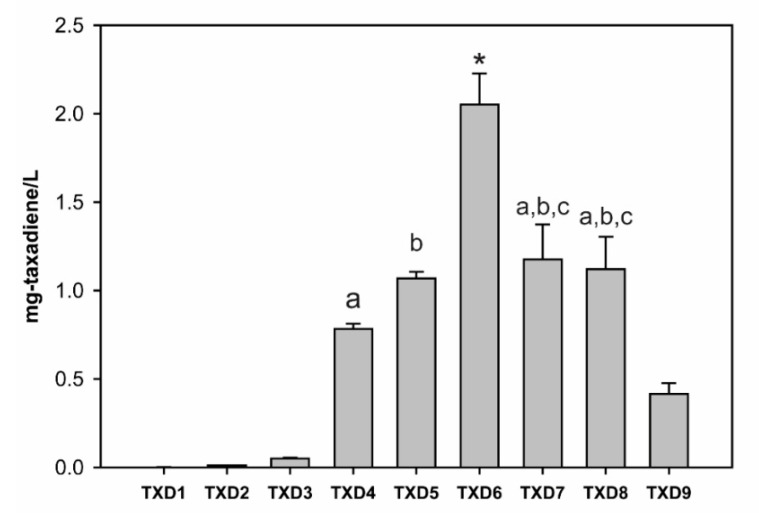
Taxadiene titers from the constructed yeast strains (Table 1). The asterisk shows significant differences (*p*-value < 0.01) on taxadiene titer of TXD6 with other strains. The alphabet ‘a, b, and c’ show no significant differences (*p*-value > 0.05) between the data with same alphabet (e.g., no significant differences between TXD4 and TXD7; and no significant differences between TXD4 and TXD8). Other data without symbol or alphabet have significant differences with other data with *p*-values at least less than 0.05 (most of them has *p*-values < 0.01).

**Figure 5:**
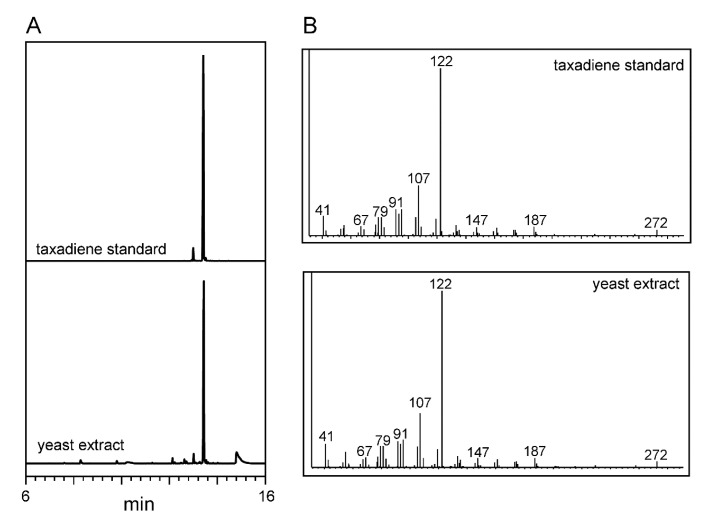
GC-MS analysis: (**A**) selected ion chromatogram and (**B**) their respective mass spectra of taxadiene standard and hexane extract from recombinant yeast (retention time 13.1 min).

Once we determined that BTS1 is the most suitable *GGPPS* to be used to produce taxadiene, we decided to use BTS1 to optimize the taxadiene production in yeast. The summary of this optimization, respective chromatogram, and the strains created in this study are shown in Figure 4 and Figure 5, and Table 1, respectively. When wild-type yeast (BY4742) was transformed with a plasmid containing a mature version (60 amino acids truncation [23]) of *Taxus media* TS (TXD1 strain), it produced 1.1 ± 0.1 μg/L of taxadiene. When TXD1 was transformed with pIPP (TXD2 strain), the production of taxadiene increased about nine-fold to 10.2 ± 0.7 μg/L. This result indicated that increasing the expression of metabolic genes in the MVA pathway can increase taxadiene production in yeast by providing more precursors, i.e., IPP and DMAPP. However, TXD2 strain is still limited by endogenous *GGPPS* activity, as shown by TXD3 strain. In TXD3 strain, when overexpressing *TS* and *BTS1* only without pIPP plasmid, i.e., without increasing any flux towards the MVA pathway, the taxadiene production was increased by 47-fold to 50.2 ± 4.7 μg/L compared to wild-type. This result confirmed that endogenous *GGPPS* activity is a critically important rate-limiting step for taxadiene production in yeast. By increasing the flux towards the MVA pathway and overexpressing *BTS1*, we created TXD4 strain which increased the production of taxadiene to 782 ± 14 μg/L, representing a 726-fold increase. This result is higher than that obtained with the strain created in a previous study when *TS*, *GGPPS*, and *tHMGR* were overexpressed [24]. Few possible differences can affect these results. First, we used *TS* from different species (*T*. *media* vs. *T*. *chinensis*) which may have different kinetic properties. Second, our study used galactose inducible promoters for *TS* and *GGPPS* while the previous study used constitutive promoters for all genes, which can lead to differences in gene stability and expression. Third, as mentioned before we used *BTS1* as *GGPPS*, while others used *SaGGPPS*, and our study showed that overexpressing *BTS1* has a larger impact on taxadiene production than *SaGGPPS*. Lastly, we overexpressed four middle enzymes (ERG8, ERG12, ERG13, and tHMGR) in the MVA pathway, while they only overexpressed tHMGR. Although tHMGR is a rate-limiting enzyme in the MVA pathway [28], increasing the expression of three other enzymes (ERG13, ERG12, and ERG8) in the MVA pathway in addition to tHMGR, can have significant effects on terpenoid production [41].

To further increase the efficiency of taxadiene production in yeast, we optimized the expression of taxadiene synthase (*TS*) by codon-optimization for yeast expression, which is a common strategy that has been used in previous studies [24,42]. Unfortunately, this strategy (TXD5 strain) only increased taxadiene production by 1.4-fold (1.07 ± 0.04 mg/L) compared to native *TS*. We suspected that TS activity could be regulated not only by translation efficiency but also by protein stability at the post-translational level. Indeed, Apel et al. reported that TS enzyme in yeast has a low solubility, which can be increased by tagging a maltose-binding protein (MBP) in the C-terminus of TS [25]. Combining this strategy with our previous metabolic engineering resulted in TXD6 strain, in which taxadiene production increased to 2.02 ± 0.40 mg/L, nearly twice more than TXD5 (without MBP) and 1900-fold when compared to TXD1 (base strain).

### 2.4. CRISPR-Mediated Gene Integration

**Figure 6:**
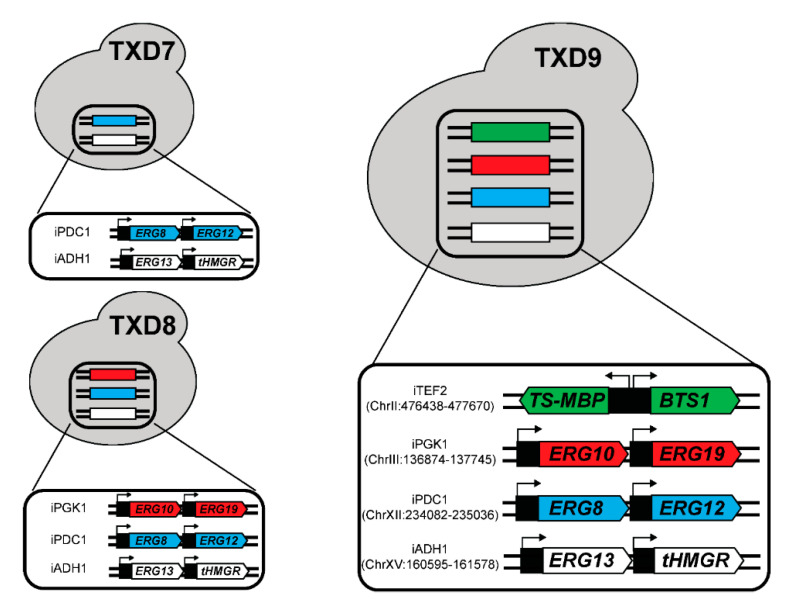
Schematic diagram of the MVA pathway genes, *BTS1*, and *TS-MBP* integration into yeast genome to create TXD7, TXD8, and TXD9. The label on the left indicates the intergenic loci of respective genes, e.g., iTEF2 means intergenic locus flanking the *TEF2* gene. The location of the loci in the genome was also indicated here. To produce taxadiene, a pESC-Ura plasmid containing *TS-MBP* and *BTS1* was transformed into TXD7 and TXD8. TXD9 is capable to produce taxadiene without plasmid. The promoters that were used were listed in Table 1.

Taxadiene is the first committed precursor of the paclitaxel biosynthesis pathway and subsequent steps are predominantly catalyzed by cytochrome P450 enzymes. Since plasmid-based gene expression is limited by available selection markers and the stability of plasmids, generating yeast strains with stable gene integration in the genome can benefit the studies of downstream candidate genes. Therefore, we sought to integrate the genes of interest into the yeast genome. Recently, we have identified several loci for strong gene expression and high integration rates by CRISPR-Cas9 [43]. We utilized four of these loci (iADH1, iPDC1, iPGK1, and iTEF2) to integrate genes in pIPP, *BTS1*, and optimized *TS-MBP* into the yeast genome (Figure 6).

First, we integrated the genes expressed from the pIPP plasmid into the yeast genome and transformed this strain with a plasmid containing *TS-MBP* and *BTS1*, creating the TXD7 strain (Figure 6). Taxadiene production in this strain was reduced by 57% to 1.18 ± 0.20 mg/L compared to TXD6. This observation can be in part explained by pIPP plasmid copy number in TXD6. A previous study showed that CEN6/ARS4-based plasmid copy number (used in pIPP) can be varied between 2–5 copies [44]. When genes in the pIPP are integrated into the genome, the total additional copy for each gene to the endogenous genes is only one. Therefore, a 57% decrease in taxadiene production by TXD7 compared to TXD6 might have been caused by the reduced copies of the MVA pathway genes in TXD7.

One investigation on the MVA pathway genes showed that *ERG19* (*mevalonate diphosphate decarboxylase*; see Figure 1) can be overexpressed to increase isoprenoid precursors while reducing sterol accumulation in yeast [45]. Another study on *ERG10* (*acetoacetyl-CoA synthase*; see Figure 1) indicated that overexpressing *ERG10* plays a role in increasing the flux towards IPP and DMAPP in yeast [46]. Therefore, we presumed that it might be necessary to overexpress all six genes in the MVA pathway, including *ERG10* and *ERG19*, to improve taxadiene production in yeast. Strain TXD8 was created by integrating these additional two genes into the yeast genome (Figure 6). However, no statistical differences (*p*-value ≥ 0.05) in taxadiene production between TXD8 (1.12 ± 0.18 mg/L) and TXD7 were observed. This result indicates that *ERG10* and *ERG19* overexpression has an insignificant impact on the flux through the MVA pathway.

Lastly, we integrated *TS-MBP* and *BTS1* into the yeast genome of the strain TXD8, resulting in the strain TXD9 (Figure 6). This strain can be grown in rich media with a high cell density to produce taxadiene. However, taxadiene production in this strain was lower (0.41 ± 0.06 mg/L) than the TXD6, which indicates that plasmid copy number, and thus gene expression levels of *TS-MBP* and *BTS1* in TXD6, play an important role in enhancing taxadiene production in yeast. As *TS-MBP* and *BTS1* were expressed from the high copy 2 μ plasmid, their single-copy genomic integration resulted in a more severe reduction in taxadiene compared to the integration of the MVA, which were expressed from a low copy CEN/ARS plasmid. The highest taxadiene-producing yeast reported so far used five copies of *GGPPS* from bacteria (*Escherichia vulneris*) and three copies of *TS* fused with different tags, which resulted in 57 mg/L taxadiene titers [26]. This strategy showed that the main bottleneck for taxadiene production in yeast is the activity of *TS* and *GGPPS*. As we integrated a single copy of *TS* and *BTS* in this work, we expect the titer of taxadiene can be further increased in our strain as we optimize the proper levels of gene expressions.

To examine the stability of transgenes in TXD9 genome, we genotyped the TXD9 strain after successive subcultures. Four independent colonies of TXD9 were grown in YP media with galactose. Then, 5% of the culture was taken and moved to fresh media every day for four days. The doubling time of BY4742 in minimal media with galactose is around 2 h [47], and thus at least 48 generations of yeast were reached in four days. After four days, genomic DNA from the four colonies were genotyped, which demonstrated that all the integrated genes are still present in the genome (Figure S2A). The production titers of taxadiene in the colonies were also measured on the first and last (4th) day, and their titers were compared (Figure S2B). The results showed that there are no significant differences (*p*-value > 0.05) in the titers between the culture from the fourth day and that from the first day, indicating that gene expressions are stable after 48 generations.

Our study shows a promising use of *BTS1* overexpression in the yeast system for taxadiene production. The TXD9 strain also provides a stable and invaluable host to further investigate the downstream pathway of the paclitaxel biosynthesis. The TXD9 strain taxadiene titer can be enhanced further by providing more copy numbers in the genome and optimizing the stability of *TS* and *BTS1*.

## 3. Materials and Methods

### 3.1. Yeast and Bacterial Strains

*Saccharomyces cerevisiae* strain BY4742 (MATα; his3Δ1; leu2Δ0; lys2Δ0; ura3Δ0) was used for transforming all taxadiene-related plasmids. *Escherichia coli* strains TOP10 were purchased from Invitrogen (Carlsbad, CA, USA) and used for cloning procedures.

### 3.2. Plasmid and Synthetic DNA Construction

The pIPP plasmid was constructed using the DNA assembler method [32]. The different DNA parts were amplified by PCR using Phusion High-Fidelity DNA polymerase (Thermo Fisher Scientific, Waltham, MA, USA), resolved by gel electrophoresis, and individually purified using Qiagen Gel Purification kit (Valencia, CA, USA). DNA parts (promoter, gene, terminator) with overlapping homologous sequences were pooled with a linearized plasmid and transformed into the appropriate auxotrophic yeast strain using the Gietz method [47]. Assembled plasmids were selected by growth on minimal medium and the resulting plasmids were recovered from yeast and transformed into *E*. *coli* for maintenance. Sanger sequencing confirmed the correct assembly of each construct. Promoters and terminators required for assembly were amplified from *S*. *cerevisiae* CEN.PK genomic DNA. Yeast mevalonate pathway genes *ERG8*, *ERG12*, *ERG13*, and a truncated *HMGR* (*tHMGR*) were also amplified from *S*. *cerevisiae* CEN.PK genomic DNA and assembled into a centromeric plasmid derived from pGREG505 [48].

*TS* and *GGPPS* expression plasmid were based on the pESC-URA plasmid (Agilent Technologies). *TS* and its modifications were cloned into MCS1 using the Gibson assembly method between *NotI* and *SpeI* sites (New England BioLabs, Ipswich, MA, USA). Meanwhile, all *GGPPS* were cloned into MCS2 using the Gibson assembly method between *XmaI* and *SalI* sites. Tags (FLAG and c-Myc, respectively) in both MCS were retained for detecting the gene expression. Synthetic genes were codon-optimized for *S*. *cerevisiae* expression by GeneArt (Thermo Fisher Scientific, Waltham, MA, USA). The linker and MBP sequences from Apel et al. were used for tagging TS with MBP [25].

### 3.3. RNA Isolation, cDNA Synthesis, and qPCR Analysis

Yeast cells were lysed with 1 mL of Trizol (Invitrogen, Carlsbad, CA, USA). Total RNA was extracted based on the manufacturer’s protocol. First-strand synthesis of cDNA was performed using 1 μg of extracted total RNA as templated mixed with the M-MulV reverse transcriptase (New England BioLabs, Ipswich, MA, USA) and anchored oligo dT_22_ (IDT, Coralville, IA, USA). The synthesis of cDNA was performed according to the manufacturer’s protocol.

For qPCR analysis, the reaction mixture was as follows: 5 μL Power SYBR Green Master Mix (Thermo Fisher Scientific, Waltham, MA, USA), 0.6 μL each of forward and reverse primers (3.35 pmol/μL), 1 μL cDNA template (250 ng/μL), and 2.8 μL of sterile H_2_O. *ROX* was used as a passive reference dye in our qPCR. qPCR was then performed using a StepOne Real-Time PCR machine (Applied Biosystems, Foster City, CA, USA). Thermocycling parameters used for qPCR were 95 °C for 10 min, 95 °C for 15 s, and a combined extension and annealing step of 60 °C for 1 min with a total of 40 cycles. Relative transcript abundance was calculated using the efficiency corrected 2^−^^∆∆^^CT^ method based on actin as the reference gene.

### 3.4. Yeast Expression and Cultivation

Yeast was transformed using the Gietz method [49] with selection on synthetic complete (SC) medium (6.7 g/L yeast nitrogen base without amino acids, 1.4 g/L appropriate synthetic drop-out mix for SC-URA, and SC-URA-LEU, and 20 g/L glucose). For taxadiene production, yeast colonies were cultivated overnight in an appropriate liquid medium at 30 °C, 200 rpm. Then, 0.6 mL of overnight culture were transferred into 30 mL medium (SC medium same as above except with 0.2 g/L glucose, 1.8 g/L galactose, and 10 mM HEPES), and were grown at 30°C, 200 rpm for 3 days. For TXD9, the colonies were grown in YPDA medium (10 g/L yeast extract, 20 g/L peptone, 40 mg/L adenine hemisulfate, 20 g/L dextrose, 0.2 g/L glucose, 1.8 g/L galactose, and 10 mM HEPES) at the same conditions as the other strains.

### 3.5. Immunoblot

Yeast cells from 30 mL three-days grown culture were harvested. Yeast crude proteins were extracted using glass beads in lysis buffer [50 mM Tris-HCl pH 7.5, 50 mM NaCl, 1 mM PMSF, protease inhibitor cocktail Complete Mini tablets (Roche Applied Science, Mannheim, Germany), and 3 μg/mL pepstatin]. Total extract was centrifuged at 14,000 rpm at 4 °C and the supernatant was collected. The concentration of total soluble protein was tested using the Bradford assay. The total soluble protein was used to run 12% SDS-PAGE followed by transfer to nitrocellulose membrane (Hybond ECL, GE Healthcare, Chicago, IL, USA) using a Tris-glycine-methanol buffer system (25 mM Tris-base, 192 mM glycine, 20% methanol). The membrane was blocked with 5% skim milk in TBST (50 mM Tris-base pH 7.6, 150 mM NaCl, and 0.05% Tween 20) for one hour at room temperature before it was incubated overnight at 4 °C with appropriate (monoclonal anti-cMyc and anti-PGK1) primary antibody (Santa Cruz Biotechnology, Dallas, TX, USA), which was diluted at 1:2000 with 5% skim milk in TBST. Subsequently, the membrane was incubated for one hour at room temperature with HRP-conjugated anti-mouse IgG secondary antibody (Santa Cruz Biotechnology, Dallas, TX, USA), which was diluted at 1:10,000 with 5% skim milk in TBST. The membrane was washed with TBST three times before it was visualized with Amersham Imager 600 (GE Healthcare, Chicago, IL, USA).

### 3.6. Yeast Metabolites Extraction and Analysis

Yeast grown in an appropriate liquid medium for three days was harvested by centrifugation at 3000 rpm for 5 min. Five milliliters of hexane were used to extract the metabolites from the yeast cells. One milliliter of the extract was obtained and analyzed using the GC-MS system with an Agilent 6890N gas chromatograph and an Agilent 5975B mass spectrometer. One microliter of the extract was injected into a DB-5MS column (30 m × 0.25 μm inner diameter × 0.25 μm film thickness) using helium as the gas carrier. Metabolites separation was programmed with an injector temperature of 280 °C and initial temperature at 100 °C, followed by increasing temperature to 300 °C at 10 °C/min and 20 °C/min to 320 °C, and the final temperature was held for 2 min. Mass detection was programmed as previously described by Engels [24]. Taxadiene standard was kindly provided by Dr. Phil Baran from the Scripps Research Institute.

### 3.7. Genes Integration

Characterized intergenic sites and the CRISPR-Cas9 platform were chosen as described previously [42]. The genes of interest were amplified and cloned into the donor DNA plasmid. Four characterized intergenic sites (iADH1, iPDC1, iTEF2, and iPGK1) were used as genome integration targets. The appropriate plasmid (with *URA3* marker) containing Cas9 genes and guide RNA (gRNA) that was specific to those sites was transformed concurrently with 1-μg linear donor DNA into yeast cells. The successful integration was confirmed with PCR from genomic DNA and the Cas9-containing plasmid was removed using 5-FOA counter-selection. The appropriate medium was used to measure taxadiene production from the genome-edited yeast strain.

## 4. Conclusions

In this study, we screened various *GGPPS* to enhance the production of taxadiene in yeast, including *GGPPS* from *Chlamydomonas reinhardtii*. Our results showed that overexpression of endogenous yeast *GGPPS* (*BTS1*) showed the highest impact on the production of taxadiene in yeast. The production of taxadiene was also optimized by boosting the flux towards the MVA pathway and by enhancing the stability and solubility of *TS* through MBP fusion, which resulted in up to 2.05 mg/L taxadiene. Finally, we constructed plasmid-free taxadiene-producing yeast strains by integrating the required taxadiene-producing genes in the yeast genome. Further optimizations, such as increasing copy numbers of *TS* and *BTS1*, fusing the *GGPPS* and *FPPS* [26,50], and reducing the flux towards squalene (ergosterol pathway) to increase the FPP pool [24] could be further carried out to enhance taxadiene production. The strain developed here can be used as a platform to elucidate the downstream genes in the paclitaxel biosynthesis pathway.

## Figures and Tables

**Table 1:** Yeast strains built in this study.

Yeast Strain	Description ^1^
TXD1	pESC-URA: Tm-TS
TXD2	pESC-URA: Tm-TS; pIPP
TXD3	pESC-URA: Tm-TS//Sc-BTS1
TXD4	pESC-URA: Tm-TS//Sc-BTS1; pIPP
TXD5	pESC-URA: Opt Tm-TS//Sc-BTS1; pIPP
TXD6	pESC-URA: Opt Tm-TS~MBP//Sc-BTS1; pIPP
TXD7	pESC-URA: Opt Tm-TS~MBP//Sc-BTS1 Integrated: iADH1: pPYK1-ERG13; pPDC1-tHMGR iPDC1: pTPI1-ERG8; pFBA1-ERG12
TXD8	pESC-URA: Opt Tm-TS~MBP//Sc-BTS1 Integrated: iADH1: pPYK1-ERG13; pPDC1-tHMGR iPDC1: pTPI1-ERG8; pFBA1-ERG12 iPGK1: pTDH3-ERG10; pTEF1-ERG19
TXD9	All integrated: iADH1: pPYK1-ERG13; pPDC1-tHMGR iPDC1: pTPI1-ERG8; pFBA1-ERG12 iPGK1: pTDH3-ERG10; pTEF1-ERG19 iTEF2: pGAL1,10-Opt Tm-TS~MBP-ScBTS1

^1^*Abbreviation*: Tm-TS, mature *Taxus media* taxadiene synthase; Sc-BTS1, *Saccharomyces cerevisiae* geranylgeranyl pyrophosphate synthase; Opt Tm-TS, codon-optimized taxadiene synthase for yeast; Opt Tm-TS~MBP, opt Tm-TS tagged in C terminus with maltose-binding protein; pGAL1,10, *GAL1* and *GAL10* bidirectional yeast promoters; pPYK1, pPDC1, pTPI1, and pFBA1, constitutive promoters that were used for pIPP (Figure 2); pTEF1 and pTDH3, promoter of yeast *TEF1* and *TDH3* genes (constitutive); iADH1, iPDC1, iPGK1, iTEF2, previously characterized intergenic loci of *ADH1*, *PDC1*, *PGK1*, and *TEF2*.

## Data Availability

The data presented in this study are available in article and supplementary material.

## Supplementary Materials

The following are available online at https://www.mdpi.com/2218-1989/11/3/147/s1, Figure S1: immunoblot data, Table S1: primers list.
