# Supplementary material for: Developing a Yeast Platform Strain for an Enhanced Taxadiene Biosynthesis by CRISPR/Cas9
Source: Metabolites. 2021 Mar 3;11(3):147. doi: 10.3390/metabo11030147 (PMC8000486; doi:10.3390/metabo11030147)
Supplement: Supplementary file 1 [file metabolites-11-00147-s001.pdf]

Supplementary Materials

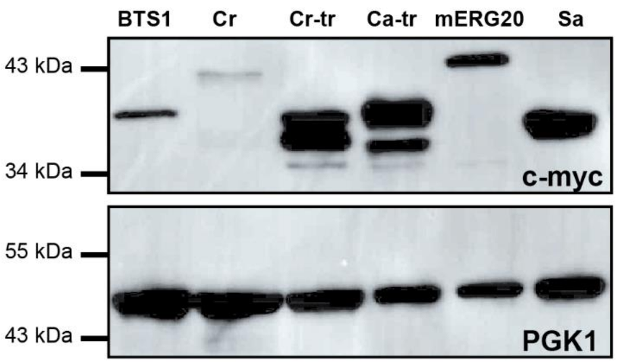

Figure S1. Immuno-blot analysis of diverse GGPP synthases expressed in yeast.

Western blot data of various GGPPSs expression, each were tagged with c-myc (top), and the expression of PGK1 for each strain were shown as control (bottom). All proteins were loaded with the same amount (5 μg).

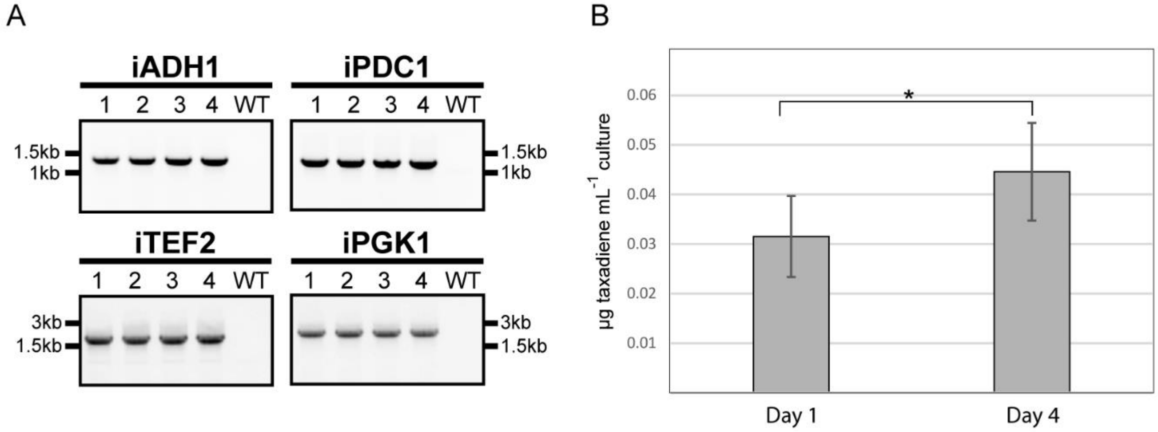

Figure S2. Stability tests of TXD9 strain after 48 generations.

A) Agarose gels of the genotyping data from four colonies of the TXD9 strain after four days of (48 generations) sub-culture. The numbers indicate genomic DNA of four independent colonies. WT is wild-type BY4742 yeast genomic DNA. The colonies with the correct gene cassette will produce bands, while no bands will be observed if the gene cassette is missing. The loci, genes, and size of positive colonies are as follow: iADH1(*ERG13*//*tHMGR*; 1.3 kb positive); iPDC1(*ERG8*//*ERG12*;1.3 kb positive); iTEF2 (*TS-MBP*//*BTS1*; 1.8 kb positive); and iPGK1(*ERG10*//*ERG19*; 2.2 kb positive). B) Taxadiene titers in the 1st subculture and the last (4th) subculture in successive 1.5 mL subcultures over 4 days. Data are means ± S.D. (n=4). Asterisk means no statistical difference (*p*-value >0.05). Note that the yields here are lower than those in Figure 4 as incubation time was shorter (24-hr) and culture volume was smaller (1.5-mL) in this experiments.

Table S1. Primers used in this study

Primers name	Sequences
	**Construction of pIPP plasmid**
506 FBA1p F	TAACCCTCACTAAAGGGAACAAAAGCTGGAGCTCGTTTAAACGGCGCGCCATCCAACTGGCACCGCTGGCTTG
ERG8 FBA1p R	CACTGAAGGCTCTCAACTCTGACATTGTTTTTATGTATTACTTGGTTATGGTTA
FBA1p ERG8 F	TATAACCATAACCAAGTAATACATAAAAACAATGTCAGAGTTGAGAGCCTTCAGTGC
CYC1t ERG8 R	AAGCGTGACATAACTAATTACATGATTATTTATCAAGATAAGTTTCCGGATC
ERG8 CYC1t F	AAGATCCGGAAACTTATCTTGATAAATAATCATGTAATTAGTTATGTCACGC
TPIp CYC1t R	TATCCGTAATCTTTAAACAGCTAGTGCAAATTAAAGCCTTCGAGCGTCCC
CYC1t TPIp F	TTTTGGGACGCTCGAAGGCTTTAATTTGCACTAGCTGTTTAAAGATTACGGA
ERG12-TPIp R	CGGTGCAGAAGTTAAGAACGGTAATGACATTGTTTTTTTATGTATGTGTTTTTTGTAGTTATAGATTTAAGC
TPIp-ERG12 F2	TCTATAACTACAAAAAACACATACATAAAAAAACAATGTCATTACCGTTCTTAACTTCTG
ADH1t-ERG12 R	CTTGACCAAACCTCTGGCGAAGAAGTCCATTATGAAGTCCATGGTAAATTCGTGTTTCC
ERG12-ADH1t F	CACGAATTTACCATGGACTTCATAATGGACTTCTTCGCCAGAGGTTTGGTC
PYK1p ADH1t R	GAGATTAATCTCCAAAATAGTAGCATTGCATGCCGGTAGAGGTGTGGTCAAT
ADH1t PYK1p F	CGCTCTTATTGACCACACCTCTACCGGCATGCAATGCTACTATTTTGGAGATT
ERG13 PYK1p R	GTTTAGTTGAGAGTTTCATTGTTTTGATGTTTTATTTGTTTTGATTGGTGTC
PYK1p ERG13 F	AAAACAAATAAAACATCAAAACAATGAAACTCTCAACTAAACTTTGTTGGTG
PGIt ERG13 R	CTTTAGGTATATATTTAAGAGCGATTTGTTTTATTTTTTAACATCGTAAGATC
ERG13 PGIt F	AGAAGATCTTACGATGTTAAAAAATAAAACAAATCGCTCTTAAATATATACC
PDC1p PGIt R	GCGGAACATATGCTCACCCAGTCGCATGTGGTATACTGGAGGCTTCATGAGT
PGIt PDC1p F	GGACATAACTCATGAAGCCTCCAGTATACCACATGCGACTGGGTGAGCATAT
tHMGR PDC1p R	TTTATTGGTTAAAACCATTGTTTTTGATTTGACTGTGTTATTTTGCGTGAGG
PDC1p tHMGR F	AAATAACACAGTCAAATCAAAAACAATGGTTTTAACCAATAAAACAGTCATT
pADH2t tHMGR R	AATCGTAAAGACATAAGAGATCCGCTTAGGATTTAATGCAGGTGACGGACC
tHMGR ADH2t F	GGTCCGTCACCTGCATTAAATCCTAAGCGGATCTCTTATGTCTTTACGAT
ADH2t 505 R	TAAGCTACTATGAAAGACTTTACAAAGAATATAGAATTATATAACTTGATGAGATGAGATGAGTAAATGACAGAAGAATTA
	**Construction of TS-MBP, BTS1, and GGPPS plasmids**

TmTS F	AGCTCGAATTCACCATGAGCAGTAGCACTGGCACTAGC
TmTS R	CTGCGACTAGTTCATACTTGAATTGGATCAATATAAACTTTTCTTATATAATCC
BTS1 F	CTATAGGGCCCGGGCGTCGAGAACATGGAGGCCAAGATAGATGAGCTG
BTS1 R	CTTCTGTTCCATGTCGAGCAATTCGGATAAGTGGTCTATTATATATAACAATTC
CaGGPPS F	CAAGGAGAAAAAACCCCGGATCCAACATGGCTACTACCAAAGAGGACGAAAG
CaGGPPS R	CTTCTGTTCCATGTCGACGCCCGGGCCATTGTCAC
CrGGPPS F	CAAGGAGAAAAAACCCCGGATCCAACATGCAAATGC
TrCrGGPPS F	CAAGGAGAAAAAACCCCGGATCCAACATGGCTCAAGTTGCTACTGCTACCG
Cr&TrCrGGPPS R	CTTCTGTTCCATGTCGACGCCCGGGCCATTTTGCC
SaGGPPS F	CAAGGAGAAAAAACCCCGGATCCAACATGTCCTACTTCG
SaGGPPS R	CTTCTGTTCCATGTCGACGCCCGGGCCTTTACGC
mERG20 F	CAAGGAGAAAAAACCCCGGATCCAACATGGCCTCCGAAAAAGAAATC
mERG20 R	CTTCTGTTCCATGTCGACGCCCGGGAGCTTGGATCTCTTGTAGACCTTGTTC
OptTS F	CAACCCTCACTAAAGGGCGGCCGCACAATGTCATCTTCTACTGGTACTTC
OptTS R	CATCCTTGTAATCCATCGATACTAGTCTAACCTGGATTGGGTCAATG
TS-MBP link F	CAATTTTAGAACCACCGCCACCACCACCAACCTGGATTGGGTCAATG
MBP F	GGTGGTGGTGGCGGTGGTTCTAAAATTG
MBP R	CATCCTTGTAATCCATCGATACTAGTCTCTTGGTAATTCTAGTTTGGGCATC

Primers for DNA integration

ERG8 & ERG12 F	GATAGGTAAATAAACGCGGATCCATCCAACTGGCACCGCTG
ERG8 & ERG12 R	CTTCATGTAATAAACACACGGATCCGCATGCCGGTAGAGGTG
ERG13 & tHMGR F	GAGCGCGGAGGGGTGTCTGAATGCTACTATTTTGGAGATTAATCTCAGTAC
ERG13 & tHMGR R	CAATGCTAGTAGAGAAGGGATCCTAGAATTATATAACTTGATGAGATGAGATGAG
ERG10-tADH R	TCCTCTATTGTCATTGAAAAGATATGAACTTCTAAATAAGCGAATTTCTTATGATTTATG
tADH1-ERG10 F	TAAATCATAAGAAATTCGCTTATTTAGAAGTTCATATCTTTTCAATGACAATAGAGGAAG
ERG10-pGAl1 R	GTAAGAATTTTTGAAAATTCGAATTCAACATGTCTCAGAACGTTTACATTGTATC
pGAL1-ERG10 F	GATACAATGTAAACGTTCTGAGACATGTTGAATTCGAATTTTCAAAAATTCTTAC
pGAL1,10 R	GTAACGGATGCTGTGTAAACGGTCATGTTTTTTCTCCTTGACGTTAAAGTATAGAG
pGAL10-ERG19 F	CTCTATACTTTAACGTCAAGGAGAAAAAACATGACCGTTTACACAGCATCCGTTAC

tCYC1-ERG19 R	CTTTTCGGTTAGAGCGGATCTTATTCCTTTGGTAGACCAGTCTTTG
ERG19-tCYC1 F	CAAAGACTGGTCTACCAAAGGAATAAGATCCGCTCTAACCGAAAAG
TS-MBP + BTS1 F	CCTATATTCCACCATAACATCAATCATGCGAGCGACCTCATGCTATACCTG
TS-MBP + BTS1 R	GTAAATACACCAGCAAGAATT CTTCGAGCGTCCCAAAACCTTC

>pIPP plasmid sequence

```
CGGTACCTAATAACTTCGTATAGCATACATTATACGAAGTTATATTAAGGGTTCTCGACGTTTTCGACACTGGATGG
CGGCGTTAGTATCGAATCGACAGCAGTATAGCGACCAGCATTCACATACGATTGACGCATGATATTACTTTCTGCGC
ACTTAACTTCGCATCTGGGCAGATGATGTCGAGGCGAAAAAAAATATAAATCACGCTAACATTTGATTAAAATAGAA
CAACTACAATATAAAAAAACTATACAAATGACAAGTTCTTGAAAACAAGAATCTTTTTATTGTCAGTACTGATTAGA
AAAACTCATCGAGCATCAAATGAAACTGCAATTTATTCATATCAGGATTATCAATACCATATTTTTGAAAAAGCCGT
TTCTGTAATGAAGGAGAAAACTCACCGAGGCAGTTCCATAGGATGGCAAGATCCTGGTATCGGTCTGCGATTCCGAC
TCGTCCAACATCAATACAACCTATTAATTTCCCCTCGTCAAAAATAAGGTTATCAAGTGAGAAATCACCATGAGTGA
CGACTGAATCCGGTGAGAATGGCAAAAGCTTATGCATTTCTTTCCAGACTTGTTCAACAGGCCAGCCATTACGCTCG
TCATCAAAATCACTCGCATCAACCAAACCGTTATTCATTCGTGATTGCGCCTGAGCGAGACGAAATACGCGATCGCT
GTTAAAAGGACAATTACAAACAGGAATCGAATGCAACCGGCGCAGGAACACTGCCAGCGCATCAACAATATTTTCAC
CTGAATCAGGATATTCTTCTAATACCTGGAATGCTGTTTTGCCGGGGATCGCAGTGGTGAGTAACCATGCATCATCA
GGAGTACGGATAAAATGCTTGATGGTCGGAAGAGGCATAAATTCCGTCAGCCAGTTTAGTCTGACCATCTCATCTGT
AACATCATTGGCAACGCTACCTTTGCCATGTTTCAGAAACAACTCTGGCGCATCGGGCTTCCCATACAATCGATAGA
TTGTCGCACCTGATTGCCCGACATTATCGCGAGCCCATTTATACCCATATAAATCAGCATCCATGTTGGAATTTAAT
CGCGGCCTCGAAACGTGAGTCTTTTCCTTACCCATGGTTGTTTATGTTCGGATGTGATGTGAGAACTGTATCCTAGC
AAGATTTTAAAAGGAAGTATATGAAAGAAGAACCTCAGTGGCAAATCCTAACCTTTTATATTTCTCTACAGGGGCGC
GGCGTGGGGACAATTCAACGCGTCTGTGAGGGGAGCGTTTCCCTGCTCGCAGGTCTGCAGCGAGGAGCCGTAATTTT
TGCTTCGCGCCGTGCGGCCATCAAAATGTATGGATGCAAATGATTATACATGGGGATGTATGGGCTAAATGTACGGG
CGACAGTCACATCATGCCCCTGAGCTGCGCACGTCAAGACTGTCAAGGAGGGTATTCTGGGCCTCCATGTCGCTGGC
CGGGTGACCCGGCGGGGACGAGGCAAGCTAAACAGATCTCTAGACCTAATAACTTCGTATAGCATACATTATACGAA
GTTATATTAAGGGTTGTCTTAATTAAGGGTACCCAATTCGCCCTATAGTGAGTCGTATTACGCGCGCTCACTGGCCG
TCGTTTTACAACGTCGTGACTGGGAAAACCCTGGCGTTACCCAACTTAATCGCCTTGCAGCACATCCCCCTTTCGCC
AGCTGGCGTAATAGCGAAGAGGCCCGCACCGATCGCCCTTCCCAACAGTTGCGCAGCCTGAATGGCGAATGGCGCGA
CGCGCCCTGTAGCGGCGCATTAAGCGCGGCGGGTGTGGTGGTTACGCGCAGCGTGACCGCTACACTTGCCAGCGCCC
TAGCGCCCGCTCCTTTCGCTTTCTTCCCTTCCTTTCTCGCCACGTTCGCCGGCTTTCCCCGTCAAGCTCTAAATCGG
GGGCTCCCTTTAGGGTTCCGATTTAGTGCTTTACGGCACCTCGACCCCAAAAAACTTGATTAGGGTGATGGTTCACG
TAGTGGGCCATCGCCCTGATAGACGGTTTTTCGCCCTTTGACGTTGGAGTCCACGTTCTTTAATAGTGGACTCTTGT
TCCAAACTGGAACAACACTCAACCCTATCTCGGTCTATTCTTTTGATTTATAAGGGATTTTGCCGATTTCGGCCTAT
TGGTTAAAAAATGAGCTGATTTAACAAAAATTTAACGCGAATTTTAACAAAATATTAACGTTTACAATTTCCTGATG
CGGTATTTTCTCCTTACGCATCTGTGCGGTATTTCACACCGCATATCGACGGTCGAGGAGAACTTCTAGTATATCCA
CATACCTAATATTATTGCCTTATTAAAAATGGAATCCCAACAATTACATCAAAATCCACATTCTCTTCAAAATCAAT
TGTCCTGTACTTCCTTGTTCATGTGTGTTCAAAAACGTTATATTTATAGGATAATTATACTCTATTTCTCAACAAGT
AATTGGTTGTTTGGCCGAGCGGTCTAAGGCGCCTGATTCAAGAAATATCTTGACCGCAGTTAACTGTGGGAATACTC
AGGTATCGTAAGATGCAAGAGTTCGAATCTCTTAGCAACCATTATTTTTTTCCTCAACATAACGAGAACACACAGGG
GCGCTATCGCACAGAATCAAATTCGATGACTGGAAATTTTTTGTTAATTTCAGAGGTCGCCTGACGCATATACCTTT
TTCAACTGAAAAATTGGGAGAAAAAGGAAAGGTGAGAGGCCGGAACCGGCTTTTCATATAGAATAGAGAAGCGTTCA
TGACTAAATGCTTGCATCACAATACTTGAAGTTGACAATATTATTTAAGGACCTATTGTTTTTTCCAATAGGTGGTT
AGCAATCGTCTTACTTTCTAACTTTTCTTACCTTTTACATTTCAGCAATATATATATATATTTCAAGGATATACCAT
TCTAATGTCTGCCCCTATGTCTGCCCCTAAGAAGATCGTCGTTTTGCCAGGTGACCACGTTGGTCAAGAAATCACAG
CCGAAGCCATTAAGGTTCTTAAAGCTATTTCTGATGTTCGTTCCAATGTCAAGTTCGATTTCGAAAATCATTTAATT
GGTGGTGCTGCTATCGATGCTACAGGTGTCCCACTTCCAGATGAGGCGCTGGAAGCCTCCAAGAAGGTTGATGCCGT
TTTGTTAGGTGCTGTGGGTGGTCCTAAATGGGGTACCGGTAGTGTTAGACCTGAACAAGGTTTACTAAAAATCCGTA
```

AAGAACTTCAATTGTACGCCAACTTAAGACCATGTAACTTTGCATCCGACTCTCTTTTAGACTTATCTCCAATCAAG
CCACAATTTGCTAAAGGTACTGACTTCGTTGTTGTCAGAGAATTAGTGGGAGGTATTTACTTTGGTAAGAGAAAGGA
AGACGATGGTGATGGTGTCGCTTGGGATAGTGAACAATACACCGTTCCAGAAGTGCAAAGAATCACAAGAATGGCCG
CTTTCATGGCCCTACAACATGAGCCACCATTGCCTATTTGGTCCTTGGATAAAGCTAATGTTTTGGCCTCTTCAAGA
TTATGGAGAAAAACTGTGGAGGAAACCATCAAGAACGAATTCCCTACATTGAAGGTTCAACATCAATTGATTGATTC
TGCCGCCATGATCCTAGTTAAGAACCCAACCCACCTAAATGGTATTATAATCACCAGCAACATGTTTGGTGATATCA
TCTCCGATGAAGCCTCCGTTATCCCAGGTTCCTTGGGTTTGTTGCCATCTGCGTCCTTGGCCTCTTTGCCAGACAAG
AACACCGCATTTGGTTTGTACGAACCATGCCACGGTTCTGCTCCAGATTTGCCAAAGAATAAGGTTGACCCTATCGC
CACTATCTTGTCTGCTGCAATGATGTTGAAATTGTCATTGAACTTGCCTGAAGAAGGTAAGGCCATTGAAGATGCAG
TTAAAAAGGTTTTGGATGCAGGTATCAGAACTGGTGATTTAGGTGGTTCCAACAGTACCACCGAAGTCGGTGATGCT
GTCGCCGAAGAAGTTAAGAAAATCCTTGCTTAAAAAGATTCTCTTTTTTTATGATATTTGTACATAAACTTTATAAA
TGAAATTCATAATAGAAACGACACGAAATTACAAAATGGAATATGTTCATAGGGTAGACGAAACTATATACGCAATC
TACATACATTTATCAAGAAGGAGAAAAAGGAGGATAGTAAAGGAATACAGGTAAGCAAATTGATACTAATGGCTCAA
CGTGATAAGGAAAAAGAATTGCACTTTAACATTAATATTGACAAGGAGGAGGGCACCACACAAAAAGTTAGGTGTAA
CAGAAAATCATGAAACTACGATTCCTAATTTGATATTGGAGGATTTTCTCTAAAAAAAAAAAAATACAACAAATAAA
AAACACTCAATGACCTGACCATTTGATGGAGTTTAAGTCAATACCTTCTTGAACCATTTCCCATAATGGTGAAAGTT
CCCTCAAGAATTTTACTCTGTCAGAAACGGCCTTACGACGTAGTCGATATGGTGCACTCTCAGTACAATCTGCTCTG
ATGCCGCATAGTTAAGCCAGCCCCGACACCCGCCAACACCCGCTGACGCGCCCTGACGGGCTTGTCTGCTCCCGGCA
TCCGCTTACAGACAAGCTGTGACCGTCTCCGGGAGCTGCATGTGTCAGAGGTTTTCACCGTCATCACCGAAACGCGC
GAGACGAAAGGGCCTCGTGATACGCCTATTTTTATAGGTTAATGTCATGATAATAATGGTTTCTTAGGACGGATCGC
TTGCCTGTAACTTACACGCGCCTCGTATCTTTTAATGATGGAATAATTTGGGAATTTACTCTGTGTTTATTTATTTT
TATGTTTTGTATTTGGATTTTAGAAAGTAAATAAAGAAGGTAGAAGAGTTACGGAATGAAGAAAAAAAAATAAACAA
AGGTTTAAAAAATTTCAACAAAAAGCGTACTTTACATATATATTTATTAGACAAGAAAAGCAGATTAAATAGATATA
CATTCGATTAACGATAAGTAAAATGTAAAATCACAGGATTTTCGTGTGTGGTCTTCTACACAGACAAGATGAAACAA
TTCGGCATTAATACCTGAGAGCAGGAAGAGCAAGATAAAAGGTAGTATTTGTTGGCGATCCCCCTAGAGTCTTTTAC
ATCTTCGGAAAACAAAAACTATTTTTTCTTTAATTTCTTTTTTTACTTTCTATTTTTAATTTATATATTTATATTAA
AAAATTTAAATTATAATTATTTTTATAGCACGTGATGAAAAGGACCCAGGTGGCACTTTTCGGGGAAATGTGCGCGG
AACCCCTATTTGTTTATTTTTCTAAATACATTCAAATATGTATCCGCTCATGAGACAATAACCCTGATAAATGCTTC
AATAATATTGAAAAAGGAAGAGTATGAGTATTCAACATTTCCGTGTCGCCCTTATTCCCTTTTTTGCGGCATTTTGC
CTTCCTGTTTTTGCTCACCCAGAAACGCTGGTGAAAGTAAAAGATGCTGAAGATCAGTTGGGTGCACGAGTGGGTTA
CATCGAACTGGATCTCAACAGCGGTAAGATCCTTGAGAGTTTTCGCCCCGAAGAACGTTTTCCAATGATGAGCACTT
TTAAAGTTCTGCTATGTGGCGCGGTATTATCCCGTATTGACGCCGGGCAAGAGCAACTCGGTCGCCGCATACACTAT
TCTCAGAATGACTTGGTTGAGTACTCACCAGTCACAGAAAAGCATCTTACGGATGGCATGACAGTAAGAGAATTATG
CAGTGCTGCCATAACCATGAGTGATAACACTGCGGCCAACTTACTTCTGACAACGATCGGAGGACCGAAGGAGCTAA
CCGCTTTTTTGCACAACATGGGGGATCATGTAACTCGCCTTGATCGTTGGGAACCGGAGCTGAATGAAGCCATACCA
AACGACGAGCGTGACACCACGATGCCTGTAGCAATGGCAACAACGTTGCGCAAACTATTAACTGGCGAACTACTTAC
TCTAGCTTCCCGGCAACAATTAATAGACTGGATGGAGGCGGATAAAGTTGCAGGACCACTTCTGCGCTCGGCCCTTC
CGGCTGGCTGGTTTATTGCTGATAAATCTGGAGCCGGTGAGCGTGGGTCTCGCGGTATCATTGCAGCACTGGGGCCA
GATGGTAAGCCCTCCCGTATCGTAGTTATCTACACGACGGGGAGTCAGGCAACTATGGATGAACGAAATAGACAGAT
CGCTGAGATAGGTGCCTCACTGATTAAGCATTGGTAACTGTCAGACCAAGTTTACTCATATATACTTTAGATTGATT
TAAAACTTCATTTTTAATTTAAAAGGATCTAGGTGAAGATCCTTTTTGATAATCTCATGACCAAAATCCCTTAACGT
GAGTTTTCGTTCCACTGAGCGTCAGACCCCGTAGAAAAGATCAAAGGATCTTCTTGAGATCCTTTTTTTCTGCGCGT
AATCTGCTGCTTGCAAACAAAAAAACCACCGCTACCAGCGGTGGTTTGTTTGCCGGATCAAGAGCTACCAACTCTTT
TTCCGAAGGTAACTGGCTTCAGCAGAGCGCAGATACCAAATACTGTCCTTCTAGTGTAGCCGTAGTTAGGCCACCAC
TTCAAGAACTCTGTAGCACCGCCTACATACCTCGCTCTGCTAATCCTGTTACCAGTGGCTGCTGCCAGTGGCGATAA
GTCGTGTCTTACCGGGTTGGACTCAAGACGATAGTTACCGGATAAGGCGCAGCGGTCGGGCTGAACGGGGGGTTCGT
GCACACAGCCCAGCTTGGAGCGAACGACCTACACCGAACTGAGATACCTACAGCGTGAGCTATGAGAAAGCGCCACG
CTTCCCGAAGGGAGAAAGGCGGACAGGTATCCGGTAAGCGGCAGGGTCGGAACAGGAGAGCGCACGAGGGAGCTTCC
AGGGGGAAACGCCTGGTATCTTTATAGTCCTGTCGGGTTTCGCCACCTCTGACTTGAGCGTCGATTTTTGTGATGCT
CGTCAGGGGGGCGGAGCCTATGGAAAAACGCCAGCAACGCGGCCTTTTTACGGTTCCTGGCCTTTTGCTGGCCTTTT
GCTCACATGTTCTTTCCTGCGTTATCCCCTGATTCTGTGGATAACCGTATTACCGCCTTTGAGTGAGCTGATACCGC
TCGCCGCAGCCGAACGACCGAGCGCAGCGAGTCAGTGAGCGAGGAAGCGGAAGAGCGCCCAATACGCAAACCGCCTC
TCCCCGCGCGTTGGCCGATTCATTAATGCAGCTGGCACGACAGGTTTCCCGACTGGAAAGCGGGCAGTGAGCGCAAC

GCAATTAATGTGAGTTACCTCACTCATTAGGCACCCCAGGCTTTACACTTTATGCTTCCGGCTCCTATGTTGTGTGG
AATTGTGAGCGGATAACAATTTCACACAGGAAACAGCTATGACCATGATTACGCCAAGCGCGCAATTAACCCTCACT
AAAGGGAACAAAAGCTGGAGCTCGTTTAAACGGCGCGCCATCCAACTGGCACCGCTGGCTTGAACAACAATACCAGC
CTTCCAACTTCTGTAAATAACGGCGGTACGCCAGTGCCACCAGTACCGTTACCTTTCGGTATACCTCCTTTCCCCAT
GTTTCCAATGCCCTTCATGCCTCCAACGGCTACTATCACAAATCCTCATCAAGCTGACGCAAGCCCTAAGAAATGAA
TAACAATACTGACAGTACTAAATAATTGCCTACTTGGCTTCACATACGTTGCATACGTCGATATAGATAATAATGAT
AATGACAGCAGGATTATCGTAATACGTAATAGTTGAAAATCTCAAAAATGTGTGGGTCATTACGTAAATAATGATAG
GAATGGGATTCTTCTATTTTTCCTTTTTCCATTCTAGCAGCCGTCGGGAAAACGTGGCATCCTCTCTTTCGGGCTCA
ATTGGAGTCACGCTGCCGTGAGCATCCTCTCTTTCCATATCTAACAACTGAGCACGTAACCAATGGAAAAGCATGAG
CTTAGCGTTGCTCCAAAAAAGTATTGGATGGTTAATACCATTTGTCTGTTCTCTTCTGACTTTGACTCCTCAAAAAA
AAAAAATCTACAATCAACAGATCGCTTCAATTACGCCCTCACAAAAACTTTTTTCCTTCTTCTTCGCCCACGTTAAA
TTTTATCCCTCATGTTGTCTAACGGATTTCTGCACTTGATTTATTATAAAAAGACAAAGACATAATACTTCTCTATC
AATTTCAGTTATTGTTCTTCCTTGCGTTATTCTTCTGTTCTTCTTTTTCTTTTGTCATATATAACCATAACCAAGTA
ATACATAAAAACAATGTCAGAGTTGAGAGCCTTCAGTGCCCCAGGGAAAGCGTTACTAGCTGGTGGATATTTAGTTT
TAGATCCGAAATATGAAGCATTTGTAGTCGGATTATCGGCAAGAATGCATGCTGTAGCCCATCCTTACGGTTCATTG
CAAGAGTCTGATAAGTTTGAAGTGCGTGTGAAAAGTAAACAATTTAAAGATGGGGAGTGGCTGTACCATATAAGTCC
TAAAACTGGCTTCATTCCTGTTTCGATAGGCGGATCTAAGAACCCTTTCATTGAAAAAGTTATCGCTAACGTATTTA
GCTACTTTAAGCCTAACATGGACGACTACTGCAATAGAAACTTGTTCGTTATTGATATTTTCTCTGATGATGCCTAC
CATTCTCAGGAGGACAGCGTTACCGAACATCGTGGCAACAGAAGATTGAGTTTTCATTCGCACAGAATTGAAGAAGT
TCCCAAAACAGGGCTGGGCTCCTCGGCAGGTTTAGTCACAGTTTTAACTACAGCTTTGGCCTCCTTTTTTGTATCGG
ACCTGGAAAATAATGTAGACAAATATAGAGAAGTTATTCATAATTTATCACAAGTTGCTCATTGTCAAGCTCAGGGT
AAAATTGGAAGCGGGTTTGATGTAGCGGCGGCAGCATATGGATCTATCAGATATAGAAGATTCCCACCCGCATTAAT
CTCTAATTTGCCAGATATTGGAAGTGCTACTTACGGCAGTAAACTGGCGCATTTGGTTAATGAAGAAGACTGGAATA
TAACGATTAAAAGTAACCATTTACCTTCGGGATTAACTTTATGGATGGGCGATATTAAGAATGGTTCAGAAACAGTA
AAACTGGTCCAGAAGGTAAAAAATTGGTATGATTCGCATATGCCGGAAAGCTTGAAAATATATACAGAACTCGATCA
TGCAAATTCTAGATTTATGGATGGACTATCTAAACTAGATCGCTTACACGAGACTCATGACGATTACAGCGATCAGA
TATTTGAGTCTCTTGAGAGGAATGACTGTACCTGTCAAAAGTATCCTGAGATCACAGAAGTTAGAGATGCAGTTGCC
ACAATTAGACGTTCCTTTAGAAAAATAACTAAAGAATCTGGTGCCGATATCGAACCTCCCGTACAAACTAGCTTATT
GGATGATTGCCAGACCTTAAAAGGAGTTCTTACTTGCTTAATACCTGGTGCTGGTGGTTATGACGCCATTGCAGTGA
TTGCTAAGCAAGATGTTGATCTTAGGGCTCAAACCGCTGATGACAAAAGATTTTCTAAGGTTCAATGGCTGGATGTA
ACTCAGGCTGACTGGGGTGTTAGGAAAGAAAAAGATCCGGAAACTTATCTTGATAAATAATCATGTAATTAGTTATG
TCACGCTTACATTCACGCCCTCCCCCCACATCCGCTCTAACCGAAAAGGAAGGAGTTAGACAACCTGAAGTCTAGGT
CCCTATTTATTTTTTTTAATAGTTATGTTAGTATTAAGAACGTTATTTATATTTCAAATTTTTCTTTTTTTTCTGTA
CAAACGCGTGTACGCATGTAACATTATACTGAAAACCTTGCTTGAGAAGGTTTTGGGACGCTCGAAGGCTTTAATTT
GCACTAGCTGTTTAAAGATTACGGATATTTAACTTACTTAGAATAATGCCATTTTTTTGAGTTATAATAATCCTACG
TTAGTGTGAGCGGGATTTAAACTGTGAGGACCTTAATACATTCAGACACTTCTGCGGTATCACCCTACTTATTCCCT
TCGAGATTATATCTAGGAACCCATCAGGTTGGTGGAAGATTACCCGTTCTAAGACTTTTCAGCTTCCTCTATTGATG
TTACACCTGGACACCCCTTTTCTGGCATCCAGTTTTTAATCTTCAGTGGCATGTGAGATTCTCCGAAATTAATTAAA
GCAATCACACAATTCTCTCGGATACCACCTCGGTTGAAACTGACAGGTGGTTTGTTACGCATGCTAATGCAAAGGAG
CCTATATACCTTTGGCTCGGCTGCTGTAACAGGGAATATAAAGGGCAGCATAATTTAGGAGTTTAGTGAACTTGCAA
CATTTACTATTTTCCCTTCTTACGTAAATATTTTTCTTTTTAATTCTAAATCAATCTTTTTCAATTTTTTGTTTGTA
TTCTTTTCTTGCTTAAATCTATAACTACAAAAAACACATACATAAAAAAACAATGTCATTACCGTTCTTAACTTCTG
CACCGGGAAAGGTTATTATTTTTGGTGAACACTCTGCTGTGTACAACAAGCCTGCCGTCGCTGCTAGTGTGTCTGCG
TTGAGAACCTACCTGCTAATAAGCGAGTCATCTGCACCAGATACTATTGAATTGGACTTCCCGGACATTAGCTTTAA
TCATAAGTGGTCCATCAATGATTTCAATGCCATCACCGAGGATCAAGTAAACTCCCAAAAATTGGCCAAGGCTCAAC
AAGCCACCGATGGCTTGTCTCAGGAACTCGTTAGTCTTTTGGATCCGTTGTTAGCTCAACTATCCGAATCCTTCCAC
TACCATGCAGCGTTTTGTTTCCTGTATATGTTTGTTTGCCTATGCCCCCATGCCAAGAATATTAAGTTTTCTTTAAA
GTCTACTTTACCCATCGGTGCTGGGTTGGGCTCAAGCGCCTCTATTTCTGTATCACTGGCCTTAGCTATGGCCTACT
TGGGGGGGTTAATAGGATCTAATGACTTGGAAAAGCTGTCAGAAAACGATAAGCATATAGTGAATCAATGGGCCTTC
ATAGGTGAAAAGTGTATTCACGGTACCCCTTCAGGAATAGATAACGCTGTGGCCACTTATGGTAATGCCCTGCTATT
TGAAAAAGACTCACATAATGGAACAATAAACACAAACAATTTTAAGTTCTTAGATGATTTCCCAGCCATTCCAATGA
TCCTAACCTATACTAGAATTCCAAGGTCTACAAAAGATCTTGTTGCTCGCGTTCGTGTGTTGGTCACCGAGAAATTT
CCTGAAGTTATGAAGCCAATTCTAGATGCCATGGGTGAATGTGCCCTACAAGGCTTAGAGATCATGACTAAGTTAAG

TAAATGTAAAGGCACCGATGACGAGGCTGTAGAAACTAATAATGAACTGTATGAACAACTATTGGAATTGATAAGAA
TAAATCATGGACTGCTTGTCTCAATCGGTGTTTCTCATCCTGGATTAGAACTTATTAAAAATCTGAGCGATGATTTG
AGAATTGGCTCCACAAAACTTACCGGTGCTGGTGGCGGCGGTTGCTCTTTGACTTTGTTACGAAGAGACATTACTCA
AGAGCAAATTGACAGTTTCAAAAAGAAATTGCAAGATGATTTTAGTTACGAGACATTTGAAACAGACTTGGGTGGGA
CTGGCTGCTGTTTGTTAAGCGCAAAAAAATTTGAATAAAGATCTTAAAATCAAATCCCTAGTATTCCAATTATTTGAA
AATAAAACTACCACAAAGCAACAAATTGACGATCTATTATTGCCAGGAAACACGAATTTACCATGGACTTCATAATG
GACTTCTTCGCCAGAGGTTTGGTCAAGTCTCCAATCAAGGTTGTCGGCTTGTCTACCTTGCCAGAAATTTACGAAAA
GATGGAAAAGGGTCAAATCGTTGGTAGATACGTTGTTGACACTTCTAAATAAGCGAATTTCTTATGATTTATGATTT
TTATTATTAAATAAGTTATAAAAAAAAATAAGTGTATACAAATTTTAAAGTGACTCTTAGGTTTTAAAACGAAAATTC
TTATTCTTGAGTAACTCTTTCCTGTAGGTCAGGTTGCTTTCTCAGGTATAGCATGAGGTCGCTCTTATTGACCACAC
CTCTACCGGCATGCAATGCTACTATTTTGGAGATTAATCTCAGTACAAAACAATATTAAAAAGAGGTGAATTATTTT
TCCCCCCTTATTTTTTTTTTGTTAAAATTGATCCAAATGTAAATAAACAATCACAAGGAAAAAAAAAAAAAAAAAAA
AAATAGCCGCCATGACCCCGGATCGTCGGTTGTGATACGGTCAGGGTAGCGCCCTGGTCAAACTTCAGAACTAAAAA
AATAATAAGGAAGAAAAAAATAGCTAATTTTTCCGGCAGAAAGATTTTCGCTACCCGAAAGTTTTTCCGGCAAGCTA
AATGGAAAAAGGAAAGATTATTGAAAGAGAAAGAAAGAAAAAAAAAAAAATGTACACCCAGACATCGGGCTTCCACAA
TTTCGGCTCTATTGTTTTCCATCTCTCGCAACGGCGGGATTCCTCTATGGCGTGTGATGTCTGTATCTGTTACTTAA
TCCAGAAACTGGCACTTGACCCAACTCTGCCACGTGGGTCGTTTTGCCATCGACAGATTGGGAGATTTTCATAGTAG
AATTCAGCATGATAGCTACGTAAATGTGTTCCGCACCGTCACAAAGTGTTTTCTACTGTTCTTTCTTCTTTCGTTCA
TTCAGTTGAGTTGAGTGAGTGCTTTGTTCAATGGATCTTAGCTAAAATGCATATTTTTTCTCTTGGTAAATGAATGC
TTGTGATGTCTTCCAAGTGATTTCCTTTCCTTCCCATATGATGCTAGGTACCTTTAGTGTCTTCCTAAAAAAAAAAA
AAGGCTCGCCATCAAAACGATATTCGTTGGCTTTTTTTTCTGAATTATAAATACTCTTTGGTAACTTTTCATTTCCA
AGAACCTCTTTTTTCCAGTTATATCATGGTCCCCTTTCAAAGTTATTCTCTACTCTTTTTCATATTCATTCTTTTTC
ATCCTTTGGTTTTTTATTCTTAACTTGTTTATTATTCTCTCTTGTTTCTATTTACAAGACACCAATCAAAACAAATA
AAACATCAAAACAATGAAACTCTCAACTAAACTTTGTTGGTGTGGTATTAAAGGAAGACTTAGGCCGCAAAAGCAAC
AACAATTACACAATACAAACTTGCAAATGACTGAACTAAAAAAACAAAAGACCGCTGAACAAAAAACCAGACCTCAA
AATGTCGGTATTAAAGGTATCCAAATTTACATCCCAACTCAATGTGTCAACCAATCTGAGCTAGAGAAATTTGATGG
CGTTTCTCAAGGTAAATACACAATTGGTCTGGGCCAAACCAACATGTCTTTTGTCAATGACAGAGAAGATATCTACT
CGATGTCCCTAACTGTTTTGTCTAAGTTGATCAAGAGTTACAACATCGACACCAACAAAATTGGTAGATTAGAAGTC
GGTACTGAAACTCTGATTGACAAGTCCAAGTCTGTCAAGTCTGTCTTGATGCAATTGTTTGGTGAAAACACTGACGT
CGAAGGTATTGACACGCTTAATGCCTGTTACGGTGGTACCAACGCGTTGTTCAACTCTTTGAACTGGATTGAATCTA
ACGCATGGGATGGTAGAGACGCCATTGTAGTTTGCGGTGATATTGCCATCTACGATAAGGGTGCCGCAAGACCAACC
GGTGGTGCCGGTACTGTTGCTATGTGGATCGGTCCTGATGCTCCAATTGTATTTGACTCTGTAAGAGCTTCTTACAT
GGAACACGCCTACGATTTTTACAAGCCAGATTTCACCAGCGAATATCCTTACGTCGATGGTCATTTTTCATTAACTT
GTTACGTCAAGGCTCTTGATCAAGTTTACAAGAGTTATTCCAAGAAGGCTATTTCTAAAGGGTTGGTTAGCGATCCC
GCTGGTTCGGATGCTTTGAACGTTTTGAAATATTTCGACTACAACGTTTTCCATGTTCCAACCTGTAAATTGGTCAC
AAAATCATACGGTAGATTACTATATAACGATTTCAGAGCCAATCCTCAATTGTTCCCAGAAGTTGACGCCGAATTAG
CTACTCGCGATTATGACGAATCTTTAACCGATAAGAACATTGAAAAAACTTTTGTTAATGTTGCTAAGCCATTCCAC
AAAGAGAGAGTTGCCCAATCTTTGATTGTTCCAACAAACACAGGTAACATGTACACCGCATCTGTTTATGCCGCCTT
TGCATCTCTATTAAACTATGTTGGATCTGACGACTTACAAGGCAAGCGTGTTGGTTTATTTTCTTACGGTTCCGGTT
TAGCTGCATCTCTATATTCTTGCAAAATTGTTGGTGACGTCCAACATATTATCAAGGAATTAGATATTACTAACAAA
TTAGCCAAGAGAATCACCGAAACTCCAAAGGATTACGAAGCTGCCATCGAATTGAGAGAAAATGCCCATTTGAAGAA
GAACTTCAAACCTCAAGGTTCCATTGAGCATTTGCAAAGTGGTGTTTACTACTTGACCAACATCGATGACAAATTTA
GAAGATCTTACGATGTTAAAAAATAAAACAAATCGCTCTTAAATATATACCTAAAGAACATTAAAGCTATATTATAA
GCAAAGATACGTAAATTTTGCTTATATTATTATACACATATCATATTTCTATATTTTTAAGATTTGGTTATATAATG
TACGTAATGCAAAGGAAATAAATTTTATACATTATTGAACAGCGTCCAAGTAACTACATTATGTGCACTAATAGTTT
AGCGTCGTGAAGACTTTATTGTGTCGCGAAAAGTAAAAATTTTAAAAATTAGAGCACCTTGAACTTGCGAAAAAGGT
TCTCATCAACTGTTTAAAAGGAGGATATCAGGTCCTATTTCTGACAAACAATATACAAATTTAGTTTCAAAGATGAA
TCAGTGCGCGAAGGACATAACTCATGAAGCCTCCAGTATACCACATGCGACTGGGTGAGCATATGTTCCGCTGATGT
GATGTGCAAGATAAACAAGCAAGGCAGAAACTAACTTCTTCTTCATGTAATAAACACACCCCGCGTTTATTTACCTA
TCTCTAAACTTCAACACCTTATATCATAACTAATATTTCTTGAGATAAGCACACTGCACCCATACCTTCCTTAAAAA
CGTAGCTTCCAGTTTTTGGTGGTTCCGGCTTCCTTCCCGATTCCGCCCGCTAAACGCATATTTTTGTTGCCTGGTGG
CATTTGCAAAATGCATAACCTATGCATTTAAAAGATTATGTATGCTCTTCTGACTTTTCGTGTGATGAGGCTCGTGG
AAAAAATGAATAATTTATGAATTTGAGAACAATTTTGTGTTGTTACGGTATTTTACTATGGAATAATCAATCAATTG

AGGATTTTATGCAAATATCGTTTGAATATTTTTCCGACCCTTTGAGTACTTTTCTTCATAATTGCATAATATTGTCC
GCTGCCCCTTTTTCTGTTAGACGGTGTCTTGATCTACTTGCTATCGTTCAACACCACCTTATTTTCTAACTATTTTT
TTTTTAGCTCATTTGAATCAGCTTATGGTGATGGCACATTTTTGCATAAACCTAGCTGTCCTCGTTGAACATAGGAA
AAAAAAATATATAAACAAGGCTCTTTCACTCTCCTTGCAATCAGATTTGGGTTTGTTCCCTTTATTTTCATATTTCT
TGTCATATTCCTTTCTCAATTATTATTTTCTACTCATAACCTCACGCAAAATAACACAGTCAAATCAAAAACAATGG
TTTTAACCAATAAAACAGTCATTTCTGGATCGAAAGTCAAAAGTTTATCATCTGCGCAATCGAGCTCATCAGGACCT
TCATCATCTAGTGAGGAAGATGATTCCCGCGATATTGAAAGCTTGGATAAGAAAATACGTCCTTTAGAAGAATTAGA
AGCATTATTAAGTAGTGGAAATACAAAACAATTGAAGAACAAAGAGGTCGCTGCCTTGGTTATTCACGGTAAGTTAC
CTTTGTACGCTTTGGAGAAAAAATTAGGTGATACTACGAGAGCGGTTGCGGTACGTAGGAAGGCTCTTTCAATTTTG
GCAGAAGCTCCTGTATTAGCATCTGATCGTTTACCATATAAAAATTATGACTACGACCGCGTATTTGGCGCTTGTTG
TGAAAATGTTATAGGTTACATGCCTTTGCCCGTTGGTGTTATAGGCCCCTTGGTTATCGATGGTACATCTTATCATA
TACCAATGGCAACTACAGAGGGTTGTTTGGTAGCTTCTGCCATGCGTGGCTGTAAGGCAATCAATGCTGGCGGTGGT
GCAACAACTGTTTTAACTAAGGATGGTATGACAAGAGGCCCAGTAGTCCGTTTCCCAACTTTGAAAAGATCTGGTGC
CTGTAAGATATGGTTAGACTCAGAAGAGGGACAAAACGCAATTAAAAAAGCTTTTAACTCTACATCAAGATTTGCAC
GTCTGCAACATATTCAAACTTGTCTAGCAGGAGATTTACTCTTCATGAGATTTAGAACAACTACTGGTGACGCAATG
GGTATGAATATGATTTCTAAAGGTGTCGAATACTCATTAAAGCAAATGGTAGAAGAGTATGGCTGGGAAGATATGGA
GGTTGTCTCCGTTTCTGGTAACTACTGTACCGACAAAAAACCAGCTGCCATCAACTGGATCGAAGGTCGTGGTAAGA
GTGTCGTCGCAGAAGCTACTATTCCTGGTGATGTTGTCAGAAAAGTGTTAAAAAGTGATGTTTCCGCATTGGTTGAG
TTGAACATTGCTAAGAATTTGGTTGGATCTGCAATGGCTGGGTCTGTTGGTGGATTTAACGCACATGCAGCTAATTT
AGTGACAGCTGTTTTCTTGGCATTAGGACAAGATCCTGCACAAAATGTTGAAAGTTCCAACTGTATAACATTGATGA
AAGAAGTGGACGGTGATTTGAGAATTTCCGTATCCATGCCATCCATCGAAGTAGGTACCATCGGTGGTGGTACTGTT
CTAGAACCACAAGGTGCCATGTTGGACTTATTAGGTGTAAGAGGCCCGCATGCTACCGCTCCTGGTACCAACGCACG
TCAATTAGCAAGAATAGTTGCCTGTGCCGTCTTGGCAGGTGAATTATCCTTATGTGCTGCCCTAGCAGCCGGCCATT
TGGTTCAAAGTCATATGACCCACAACAGGAAACCTGCTGAACCAACAAAACCTAACAATTTGGACGCCACTGATATA
AATCGTTTGAAAGATGGGTCCGTCACCTGCATTAAATCCTAAGCGGATCTCTTATGTCTTTACGATTTATAGTTTTC
ATTATCAAGTATGCCTATATTAGTATATAGCATCTTTAGATGACAGTGTTCGAAGTTTCACGAATAAAAGATAATAT
TCTACTTTTTGCTCCCACCGCGTTTGCTAGCACGAGTGAACACCATCCCTCGCCTGTGAGTTGTACCCATTCCTCTA
AACTGTAGACATGGTAGCTTCAGCAGTGTTCGTTATGTACGGCATCCTCCAACAAACAGTCGGTTATAGTTTGTCCT
GCTCCTCTGAATCGTCTCCCTCGATATTTCTCATTTTCCTTCGCATGCCAGCATTGAAATGATCGAAGTTCAATGAT
GAAACGGTAATTCTTCTGTCATTTACTCATCTCATCTCATCAAGTTATATAATTCTAGCGGCCG

pESC-URA::optT.m.TS-MBP//BTS1

optimized T.m.TS-MBP: red

BTS1: blue

TCGCGCGTTTCGGTGATGACGGTGAAAACCTCTGACACATGCAGCTCCCGGAGACGGTCACAGCTTGTCTGTAAGCG
GATGCCGGGAGCAGACAAGCCCGTCAGGGCGCGTCAGCGGGTGTTGGCGGGTGTCGGGGCTGGCTTAACTATGCGGC
ATCAGAGCAGATTGTACTGAGAGTGCACCATACCACAGCTTTTCAATTCAATTCATCATTTTTTTTTTATTCTTTTT
TTTGATTTCGGTTTCTTTGAAATTTTTTTGATTCGGTAATCTCCGAACAGAAGGAAGAACGAAGGAAGGAGCACAGA
CTTAGATTGGTATATATACGCATATGTAGTGTTGAAGAAACATGAAATTGCCCAGTATTCTTAACCCAACTGCACAG
AACAAAAACCTGCAGGAAACGAAGATAAATCATGTCGAAAGCTACATATAAGGAACGTGCTGCTACTCATCCTAGTC
CTGTTGCTGCCAAGCTATTTAATATCATGCACGAAAAGCAAACAAACTTGTGTGCTTCATTGGATGTTCGTACCACC
AAGGAATTACTGGAGTTAGTTGAAGCATTAGGTCCCAAAATTTGTTTACTAAAAACACATGTGGATATCTTGACTGA
TTTTTCCATGGAGGGCACAGTTAAGCCGCTAAAGGCATTATCCGCCAAGTACAATTTTTTACTCTTCGAAGACAGAA
AATTTGCTGACATTGGTAATACAGTCAAATTGCAGTACTCTGCGGGTGTATACAGAATAGCAGAATGGGCAGACATT
ACGAATGCACACGGTGTGGTGGGCCCAGGTATTGTTAGCGGTTTGAAGCAGGCGGCAGAAGAAGTAACAAAGGAACC
TAGAGGCCTTTTGATGTTAGCAGAATTGTCATGCAAGGGCTCCCTATCTACTGGAGAATATACTAAGGGTACTGTTG
ACATTGCGAAGAGCGACAAAGATTTTGTTATCGGCTTTATTGCTCAAAGAGACATGGGTGGAAGAGATGAAGGTTAC
GATTGGTTGATTATGACACCCGGTGTGGGTTTAGATGACAAGGGAGACGCATTGGGTCAACAGTATAGAACCGTGGA
TGATGTGGTCTCTACAGGATCTGACATTATTATTGTTGGAAGAGGACTATTTGCAAAGGGAAGGGATGCTAAGGTAG
AGGGTGAACGTTACAGAAAAGCAGGCTGGGAAGCATATTTGAGAAGATGCGGCCAGCAAAACTAAAAAACTGTATTA
TAAGTAAATGCATGTATACTAAACTCACAAATTAGAGCTTCAATTTAATTATATCAGTTATTACCCTATGCGGTGTG
AAATACCGCACAGATGCGTAAGGAGAAAATACCGCATCAGGAAATTGTAAACGTTAATATTTTGTTAAAATTCGCGT
TAAATTTTTGTTAAATCAGCTCATTTTTTAACCAATAGGCCGAAATCGGCAAAATCCCTTATAAATCAAAAGAATAG
ACCGAGATAGGGTTGAGTGTTGTTCCAGTTTGGAACAAGAGTCCACTATTAAAGAACGTGGACTCCAACGTCAAAGG
GCGAAAAACCGTCTATCAGGGCGATGGCCCACTACGTGAACCATCACCCTAATCAAGTTTTTTGGGGTCGAGGTGCC
GTAAAGCACTAAATCGGAACCCTAAAGGGAGCCCCCGATTTAGAGCTTGACGGGGAAAGCCGGCGAACGTGGCGAGA
AAGGAAGGGAAGAAAGCGAAAGGAGCGGGCGCTAGGGCGCTGGCAAGTGTAGCGGTCACGCTGCGCGTAACCACCAC
ACCCGCCGCGCTTAATGCGCCGCTACAGGGCGCGTCCATTCGCCATTCAGGCTGCGCAACTGTTGGGAAGGGCGATC
GGTGCGGGCCTCTTCGCTATTACGCCAGCTGAATTGGAGCGACCTCATGCTATACCTGAGAAAGCAACCTGACCTAC
AGGAAAGAGTTACTCAAGAATAAGAATTTTCGTTTTAAAACCTAAGAGTCACTTTAAAATTTGTATACACTTATTTT
TTTTATAACTTATTTAATAATAAAAATCATAAATCATAAGAAATTCGCTTATTTAGAAGTGTCAACAACGTATCTAC
CAACGATTTGACCCTTTTCCATCTTTTCGTAAATTTCTGGCAAGGTAGACAAGCCGACAACCTTGATTGGAGACTTG
ACCAAACCTCTGGCGAAGAATTGTTAATTAAGAGCTCAGATCTTATCGTCGTCATCCTTGTAATCCATCGATACTAG
TCTCTTGGTAATTCTAGTTTGGGCATCCTTCAAAGCTTCATCAACAGTTTGTCTACCAGAAGCAGCATTGATAACAG
CGGTTCTAACAGCATACCAAAAAGCAGACATTTGTGGGATGTTTGGCATAATCTCACCCTTTTGAGCATTTTCCATA
GTTGCAGCAATTCTTGGGTCCTTAGCCAATTCTTCTTCATAAGACTTCAAAGCAACAGCACCCAATGGCTTATCTTT
GTTAACAGCTTCCAAACCTTCGTCGGTCAACAAGTAGTTCTCCAAAAATTCTTTAGCCAGTTCTTTGTTTGGAGAAG
CAGCGTTAATACCAGCAGACAAAACACCAACGAATGGCTTAGATGGTTGACCTTTAAAAGTTGGCAAAACGGTAACA
CCGTAGTTAACCTTAGAAGTATCAATGTTAGACCAAGCCCATGGACCGTTAATAGTCATAGCAGTTTCACCCTTGTT
AAAAGCAGCTTCAGCAATAGAGTAATCGGTATCAGCGTTCATGTGCTTGTTTTTGATCAGATCAACCAAGAAAGTCA
AACCAGCTTTAGCACCAGCATTATCAACACCAACATCCTTGATGTCGTACTTGCCATTTTCGTACTTAAAAGCGTAA
CCACCATCAGCTGCAATCAATGGCCACGTGAAATATGGTTCTTGCAAGTTAAACATCAAGGCGGATTTACCTTTGGC
CTTCAATTCTTTGTCCAAAGCTGGAATTTCTTCCCAGGTTTTTGGTGGATTGGGCAACAAGTCTTTGTTGTAGATCA
AAGACAAGGCTTCAACAGCAATTGGGTAAGCAATCAATTTACCGTTGTATCTAACGGCATCCCAAGTGAATGGATAC
AACTTATCTTGGAAAGCCTTATCTGGTGTAATTTCAGCCAACAAACCAGATTGAGCATAACCACCAAATCTATCATG
AGCCCAAAAGATAATATCTGGACCATCACCAGTAGCAGCAACTTGTGGAAACTTTTCTTCCAGCTTATCTGGATGTT
CAACGGTAACCTTAATACCGGTGTCTTTTTCGAACTTCTTACCAACTTCAGCCAAACCATTATAACCCTTATCACCG
TTGATCCAGATGACCAATTTACCTTCTTCAATTTTAGAACCACCGCCACCACCACCAACCTGGATTGGGTCAATGTA
AACCTTACGGATGTAATCCTTAATCTCTTCGTTGGCAATACCGTAACCATCAATGAACTTGTAGAAGATCTGGACGC
ACAATCTCAAGTTAAAGATGAAAGATTTGCAACCCATTGGAATATCGTTGGAGGGCTTAAAGTATTCAAAAGAGGCT
TCTTTCAAGGCACGATCAACAACTCTACAGATATGTTTGATAGCGTCCTCTTCAGTAGCACCTGGATTATCCTTCAT

GTAACAAGCAATACCAGAAGCTTGTTGACCTCTAGCTTTTTCAGCTTGGTAGGTCTTGGTATCATTAGTCAATCTCC
AAGACAAAGAAACCAGCTCGAACATGTTAGATGGGTAGTGAACTTTTTCGACAACGTCATCCTTAACCAATTCACCC
ATCAACAAAATTGGCTGTAAAGTACATGGACCCAAACCAACAGAGATAGCGTAGGTTTTCAAGTACTCTTCGAAAGT
TGGAATGTAACCAGCTTCCAACCATTCACGTTCTTGAACATAGCAGTTAAAGTACAACTCCCATGGCTTTCTAATAT
GGGCCAACATATCTCTACCTTGAACCTTAACGACATCGTTGTTAACCTCTTCCATCAATTTGAACCAGACCTTAAAA
CAGGTCTGCATACATTCTGGAATTTCGTGTAACAAAGAAGTGTCCCATCTCTTAACACCTTCAGTGAAAGACTTCAA
CTCATCCAAAGTAGCGAAAATATCGGCCATATCATCGAACAAAACTTGCAAGCAACCAATCTTAGTGAAAGCAATTC
TAGTAGCGGAGTATTCTGGTTCGAAAGTAGCAGAAGAAAAATAGACTTCGGCAACTCTATGTCTGGTGAAGTTAATA
TCAGCCATACCAGATTCTTTCCACCACCTAGTCAACAACTTCAATTCTTCTTGGTGCAAGGATTGGACGATGTTGAA
ATCCAACTTAGCCAATTCCAAGCACTTAGAGTTAGACAAGGATGGCATTCTATACAAGGTCTTTCTTTGCCAGACGT
AATCATCATCGTAAGAATCGATGTAGGATCTAGCTTCCAATCTTGGAATGGACATATGCCATGGGTATTCAACAACG
TATTCGATCTCTTTGTACAGCTTGGTGTTGGTAGAAATTTTTGTGGCCAAAGCATCTCTCAAGTATGGTTCAGCAAA
TTTTCTAGCATCATCCATAGCACCTTCATCTGGAAATGCCAAATCAGAAGCTCTGAACAAGTTGACAACGGATCTCA
ATTCAACATGAGTTTGACCAGCAGAAGAGAAGAAACGACCATTTTCGTCCTTAAAGTTGTTCAAGACGTCGGAAGAA
ACATCGTAACCATGAGTTCTCAATGTTCTCAAACCCAAGGCTGTGGTATTCAAATCTGGAACCAAAGAATCTCTACC
CCAACCAATACCTCTTTCAGACCAATGTCTGTAAACGTAGTCCAAAGCAACCTTGATTTCTTGCTTGAAATGTCTAC
CGATACCTAAGTGTTCGATGTTATCAACCAAGGACAATCTTTCCAGCAAATCGATAGAGTACATGCATGGAACACAA
CCACCGAACTTATCCAACAAATTGTTCAACAGGGTGAAACACTTTTCGTCACCAGTATTCATCAAAACGCAAGCAGT
AGAAGCTGGAGATGACAAAAATGAACCGTCCTTAGATTGGAACCTCATGATCTTGTTCCAATCGATAACTTCTTCCA
AACCTTCCAAAGCGTTCAACATATTAGCTGGAATGTTATCAGCAACAGCAGAAACATCAGTCAATCTGGCTTCTCTA
GTAGTAGACAAAGACTTGATGAATGGCAGATCGTATGGCAAGTTAATACCTAAAGCCTTAGCCTTTTGCAACAAAGC
TGGGAAGATAATTTCGAAGTCTGGAGACAATTCATCCTCTTCGTTCAACAAACGCAAGTTTTCAGCAATGAACTCAG
TACCTTGTTCAACTTGAGAATGACCGGTTTTCCAAACAGACAGAGCAATAACAGAGTTAACGGTGTTCAACAACCTA
TCGCACAATGAGAAATGAGATTCAATACCCCAAGAACCATCTTGCAACTGGTTGTTCAAAACCCAATTCAAAGCTTG
TGGAAATCTTGGCTTTTCAGAACCATCAGAAGAAACAGTAGCAACTCTAGCAACCCAAGCAGTATCATAAGCAGATG
GAGAAATATCACCATCACCTAAGGCGTTGAACATATCCTTGATCTTAACAACCAATTCGTCAGCTCTTTCTTGAAAG
GTAGAAGATTCCCTGAATGGAGTTTCCAAAGTTTGGATAACGTTGTGATGCCACAAATCACCATGGTAATTAGCAGA
CAATCTTGGGATGTCATCAACGATGGTAGAAGAAGTTTCAGAAACAACCTTGGAAGTACCAGTAGAAGATGACATTG
TGCGGCCGCCCTTTAGTGAGGGTTGAATTCGAATTTTCAAAAATTCTTACTTTTTTTTTGGATGGACGCAAAGAAGT
TTAATAATCATATTACATGGCATTACCACCATATACATATCCATATACATATCCATATCTAATCTTACTTATATGTT
GTGGAAATGTAAAGAGCCCCATTATCTTAGCCTAAAAAAACCTTCTCTTTGGAACTTTCAGTAATACGCTTAACTGC
TCATTGCTATATTGAAGTACGGATTAGAAGCCGCCGAGCGGGTGACAGCCCTCCGAAGGAAGACTCTCCTCCGTGCG
TCCTCGTCTTCACCGGTCGCGTTCCTGAAACGCAGATGTGCCTCGCGCCGCACTGCTCCGAACAATAAAGATTCTAC
AATACTAGCTTTTATGGTTATGAAGAGGAAAAATTGGCAGTAACCTGGCCCCACAAACCTTCAAATGAACGAATCAA
ATTAACAACCATAGGATGATAATGCGATTAGTTTTTTAGCCTTATTTCTGGGGTAATTAATCAGCGAAGCGATGATT
TTTGATCTATTAACAGATATATAAATGCAAAAACTGCATAACCACTTTAACTAATACTTTCAACATTTTCGGTTTGT
ATTACTTCTTATTCAAATGTAATAAAAGTATCAACAAAAAATTGTTAATATACCTCTATACTTTAACGTCAAGGAGA
AAAAACCCCGGATCCGTAATACGACTCACTATAGGGCCCGGGCGTCGAGAACATGGAGGCCAAGATAGATGAGCTGA
TCAATAATGATCCTGTTTGGTCCAGCCAAAATGAAAGCTTGATTTCAAAACCTTATAATCACATCCTTTTGAAACCT
GGCAAGAACTTTAGACTAAATTTAATAGTTCAAATTAACAGAGTTATGAATTTGCCCAAAGACCAGCTGGCCATAGT
TTCGCAAATTGTTGAGCTCTTGCATAATTCCAGCCTTTTAATCGACGATATAGAAGATAATGCTCCCTTGAGAAGGG
GACAGACCACTTCTCACTTAATCTTCGGTGTACCCTCCACTATAAACACCGCAAATTATATGTATTTCAGAGCCATG
CAACTTGTATCGCAGCTAACCACAAAAGAGCCTTTGTATCATAATTTGATTACGATTTTCAACGAAGAATTGATCAA
TCTACATAGGGGACAAGGCTTGGATATATACTGGAGAGACTTTCTGCCTGAAATCATACCTACTCAGGAGATGTATT
TGAATATGGTTATGAATAAAACAGGCGGCCTTTTCAGATTAACGTTGAGACTCATGGAAGCGCTGTCTCCTTCCTCA
CACCACGGCCATTCGTTGGTTCCTTTCATAAATCTTCTGGGTATTATTTATCAGATTAGAGATGATTACTTGAATTT
GAAAGATTTCCAAATGTCCAGCGAAAAAGGCTTTGCTGAGGACATTACAGAGGGGAAGTTATCTTTTCCCATCGTCC
ACGCCCTTAACTTCACTAAAACGAAAGGTCAAACTGAGCAACACAATGAAATTCTAAGAATTCTCCTGTTGAGGACA
AGTGATAAAGATATAAAACTAAAGCTGATTCAAATACTGGAATTCGACACCAATTCATTGGCCTACACCAAAAATTT
TATTAATCAATTAGTGAATATGATAAAAAATGATAATGAAAATAAGTATTTACCTGATTTGGCTTCGCATTCCGACA
CCGCCACCAATTTACATGACGAATTGTTATATATAATAGACCACTTATCCGAATTGCTCGACATGGAACAGAAGTTG
ATTTCCGAAGAAGACCTCGAGTAAGCTTGGTACCGCGGCTAGCTAAGATCCGCTCTAACCGAAAAGGAAGGAGTTAG
ACAACCTGAAGTCTAGGTCCCTATTTATTTTTTTATAGTTATGTTAGTATTAAGAACGTTATTTATATTTCAAATTT

TTCTTTTTTTTCTGTACAGACGCGTGTACGCATGTAACATTATACTGAAAACCTTGCTTGAGAAGGTTTTGGGACGC
TCGAAGATCCAGCTGCATTAATGAATCGGCCAACGCGCGGGGAGAGGCGGTTTGCGTATTGGGCGCTCTTCCGCTTC
CTCGCTCACTGACTCGCTGCGCTCGGTCGTTCGGCTGCGGCGAGCGGTATCAGCTCACTCAAAGGCGGTAATACGGT
TATCCACAGAATCAGGGGATAACGCAGGAAAGAACATGTGAGCAAAAGGCCAGCAAAAGGCCAGGAACCGTAAAAAG
GCCGCGTTGCTGGCGTTTTTCCATAGGCTCCGCCCCCCTGACGAGCATCACAAAAATCGACGCTCAAGTCAGAGGTG
GCGAAACCCGACAGGACTATAAAGATACCAGGCGTTTCCCCCTGGAAGCTCCCTCGTGCGCTCTCCTGTTCCGACCC
TGCCGCTTACCGGATACCTGTCCGCCTTTCTCCCTTCGGGAAGCGTGGCGCTTTCTCATAGCTCACGCTGTAGGTAT
CTCAGTTCGGTGTAGGTCGTTCGCTCCAAGCTGGGCTGTGTGCACGAACCCCCCGTTCAGCCCGACCGCTGCGCCTT
ATCCGGTAACTATCGTCTTGAGTCCAACCCGGTAAGACACGACTTATCGCCACTGGCAGCAGCCACTGGTAACAGGA
TTAGCAGAGCGAGGTATGTAGGCGGTGCTACAGAGTTCTTGAAGTGGTGGCCTAACTACGGCTACACTAGAAGGACA
GTATTTGGTATCTGCGCTCTGCTGAAGCCAGTTACCTTCGGAAAAAGAGTTGGTAGCTCTTGATCCGGCAAACAAAC
CACCGCTGGTAGCGGTGGTTTTTTTGTTTGCAAGCAGCAGATTACGCGCAGAAAAAAAGGATCTCAAGAAGATCCTT
TGATCTTTTCTACGGGGTCTGACGCTCAGTGGAACGAAAACTCACGTTAAGGGATTTTGGTCATGAGATTATCAAAA
AGGATCTTCACCTAGATCCTTTTAAATTAAAAATGAAGTTTTAAATCAATCTAAAGTATATATGAGTAAACTTGGTC
TGACAGTTACCAATGCTTAATCAGTGAGGCACCTATCTCAGCGATCTGTCTATTTCGTTCATCCATAGTTGCCTGAC
TCCCCGTCGTGTAGATAACTACGATACGGGAGGGCTTACCATCTGGCCCCAGTGCTGCAATGATACCGCGAGACCCA
CGCTCACCGGCTCCAGATTTATCAGCAATAAACCAGCCAGCCGGAAGGGCCGAGCGCAGAAGTGGTCCTGCAACTTT
ATCCGCCTCCATCCAGTCTATTAATTGTTGCCGGGAAGCTAGAGTAAGTAGTTCGCCAGTTAATAGTTTGCGCAACG
TTGTTGCCATTGCTACAGGCATCGTGGTGTCACGCTCGTCGTTTGGTATGGCTTCATTCAGCTCCGGTTCCCAACGA
TCAAGGCGAGTTACATGATCCCCCATGTTGTGCAAAAAAGCGGTTAGCTCCTTCGGTCCTCCGATCGTTGTCAGAAG
TAAGTTGGCCGCAGTGTTATCACTCATGGTTATGGCAGCACTGCATAATTCTCTTACTGTCATGCCATCCGTAAGAT
GCTTTTCTGTGACTGGTGAGTACTCAACCAAGTCATTCTGAGAATAGTGTATGCGGCGACCGAGTTGCTCTTGCCCG
GCGTCAATACGGGATAATACCGCGCCACATAGCAGAACTTTAAAAGTGCTCATCATTGGAAAACGTTCTTCGGGGCG
AAAACTCTCAAGGATCTTACCGCTGTTGAGATCCAGTTCGATGTAACCCACTCGTGCACCCAACTGATCTTCAGCAT
CTTTTACTTTCACCAGCGTTTCTGGGTGAGCAAAAACAGGAAGGCAAAATGCCGCAAAAAAGGGAATAAGGGCGACA
CGGAAATGTTGAATACTCATACTCTTCCTTTTTCAATATTATTGAAGCATTTATCAGGGTTATTGTCTCATGAGCGG
ATACATATTTGAATGTATTTAGAAAAATAAACAAATAGGGGTTCCGCGCACATTTCCCCGAAAAGTGCCACCTGAAC
GAAGCATCTGTGCTTCATTTTGTAGAACAAAAATGCAACGCGAGAGCGCTAATTTTTCAAACAAAGAATCTGAGCTG
CATTTTTACAGAACAGAAATGCAACGCGAAAGCGCTATTTTACCAACGAAGAATCTGTGCTTCATTTTTGTAAAACA
AAAATGCAACGCGAGAGCGCTAATTTTTCAAACAAAGAATCTGAGCTGCATTTTTACAGAACAGAAATGCAACGCGA
GAGCGCTATTTTACCAACAAAGAATCTATACTTCTTTTTTGTTCTACAAAAATGCATCCCGAGAGCGCTATTTTTCT
AACAAAGCATCTTAGATTACTTTTTTTCTCCTTTGTGCGCTCTATAATGCAGTCTCTTGATAACTTTTTGCACTGTA
GGTCCGTTAAGGTTAGAAGAAGGCTACTTTGGTGTCTATTTTCTCTTCCATAAAAAAAGCCTGACTCCACTTCCCGC
GTTTACTGATTACTAGCGAAGCTGCGGGTGCATTTTTTCAAGATAAAGGCATCCCCGATTATATTCTATACCGATGT
GGATTGCGCATACTTTGTGAACAGAAAGTGATAGCGTTGATGATTCTTCATTGGTCAGAAAATTATGAACGGTTTCT
TCTATTTTGTCTCTATATACTACGTATAGGAAATGTTTACATTTTCGTATTGTTTTCGATTCACTCTATGAATAGTT
CTTACTACAATTTTTTTGTCTAAAGAGTAATACTAGAGATAAACATAAAAAATGTAGAGGTCGAGTTTAGATGCAAG
TTCAAGGAGCGAAAGGTGGATGGGTAGGTTATATAGGGATATAGCACAGAGATATATAGCAAAGAGATACTTTTGAG
CAATGTTTGTGGAAGCGGTATTCGCAATATTTTAGTAGCTCGTTACAGTCCGGTGCGTTTTTGGTTTTTTGAAAGTG
CGTCTTCAGAGCGCTTTTGGTTTTCAAAAGCGCTCTGAAGTTCCTATACTTTCTAGAGAATAGGAACTTCGGAATAG
GAACTTCAAAGCGTTTCCGAAAACGAGCGCTTCCGAAAATGCAACGCGAGCTGCGCACATACAGCTCACTGTTCACG
TCGCACCTATATCTGCGTGTTGCCTGTATATATATATACATGAGAAGAACGGCATAGTGCGTGTTTATGCTTAAATG
CGTACTTATATGCGTCTATTTATGTAGGATGAAAGGTAGTCTAGTACCTCCTGTGATATTATCCCATTCCATGCGGG
GTATCGTATGCTTCCTTCAGCACTACCCTTTAGCTGTTCTATATGCTGCCACTCCTCAATTGGATTAGTCTCATCCT
TCAATGCTATCATTTCCTTTGATATTGGATCATACTAAGAAACCATTATTATCATGACATTAACCTATAAAAATAGG
CGTATCACGAGGCCCTTTCGTC

>native T.m.TS (truncated)

ATGAGCAGTAGCACTGGCACTAGCAAGGTGGTTTCCGAGACTTCCAGTACCATTGTGGATGATATCCCTCGACTCTC
CGCCAATTATCATGGCGATCTGTGGCACCACAATGTTATACAAACTCTGGAGACACCATTTCGTGAGAGTTCTACTT
TCCAAGAACGGGCAGACGAGCTGGTTGTGAAAATTAAAGATATGTTCAATGCGCTCGGAGACGGAGATATCAGTCCG
TCTGCATACGACACTGCGTGGGTGGCGAGGGTGGCGACCGTTTCCTCTGATGGATCTGAGAAGCCACGGTTTCCTCA

GGCCCTCAACTGGGTTTTAAACAACCAGCTCCAAGATGGATCATGGGGTATCGAATCGCACTTTAGTTTATGCGATC
GATTGCTTAACACGGTCAATTCTGTTATCGCCCTCTCGGTTTGGAAAACAGGGCACAGCCAAGTAGAACAAGGTACT
GAGTTTATTGCAGAGAATCTAAGATTACTCAATGAGGAAGATGAGTTGTCCCCGGATTTCGAAATAATCTTTCCTGC
TCTGCTGCAAAAGGCAAAAGCGTTGGGGATCAATCTTCCTTACGATCTTCCATTTATCAAATCTTTGTCGACAACAC
GGGAAGCCAGGCTTACAGATGTTTCTGCGGCAGCAGACAATATTCCAGCCAACATGTTGAATGCGTTGGAGGGTCTG
GAGGAAGTTATTGATTGGAACAAGATTATGAGGTTTCAAAGTAAAGATGGATCTTTCCTGAGCTCCCCTGCCTCCAC
TGCCTGTGTACTGATGAATACAGGGGACGAAAAATGTTTCACTCTTCTCAACAATCTGCTGGACAAATTCGGCGGCT
GCGTGCCCTGTATGTATTCCATCGATCTGCTGGAACGCCTTTCGCTGGTTGATAACATTGAGCATCTCGGAATCGGT
CGCCATTTCAAACAAGAAATCAAAGTAGCTCTTGATTATGTCTACAGACATTGGAGTGAAAGGGGCATCGGTTGGGG
CAGAGACAGCCTTGTTCCAGATCTCAACACAACAGCCCTCGGCCTGCGAACTCTTCGCACGCACGGATACGATGTTT
CTTCAGATGTTTTGAATAATTTCAAAGATGAAAACGGGCGGTTCTTCTCCTCTGCGGGCCAAACCCATGTCGAATTG
AGAAGCGTGGTGAATCTTTTCAGAGCTTCCGACCTTGCATTTCCTGACGAAGGAGCTATGGACGATGCTAGAAAATT
TGCAGAACCATATCTTAGAGACGCACTTGCAACGAAAATCTCAACCAATACAAAACTATACAAAGAGATTGAGTACG
TGGTGGAGTACCCTTGGCACATGAGTATCCCACGCCTAGAAGCTAGAAGTTATATTGATTCGTATGACGACGATTAT
GTATGGCAGAGGAAGACTCTATACAGAATGCCATCTTTGAGTAATTCAAAATGTTTAGAATTGGCAAAATTGGACTT
CAATATCGTACAATCTTTGCATCAAGAGGAGTTGAAGCTTCTAACAAGATGGTGGAAGGAATCCGGCATGGCAGATA
TAAATTTCACTCGACACCGAGTGGCGGAGGTTTATTTTTCATCAGCTACATTTGAACCTGAATATTCTGCCACCAGA
ATTGCCTTCACAAAAATTGGTTGTTTACAAGTCCTTTTTGATGATATGGCTGACATCTTTGCAACACTAGATGAATT
GAAAAGTTTCACTGAGGGAGTAAAGAGATGGGATACATCTTTGCTACATGAGATTCCAGAGTGTATGCAAACTTGCT
TTAAAGTTTGGTTCAAATTAATGGAAGAAGTAAATAATGATGTGGTTAAGGTACAAGGACGTGACATGCTCGCTCAC
ATAAGAAAACCTTGGGAGTTGTACTTCAATTGTTACGTACAAGAAAGGGAGTGGCTTGAAGCTGGGTATATACCAAC
TTTTGAAGAGTACTTAAAGACTTATGCTATATCAGTAGGCCTTGGACCGTGTACCCTACAACCAATACTACTGATGG
GTGAGCTTGTGAAAGATGATGTTGTTGAGAAAGTGCACTATCCCTCAAATATGTTTGAGCTTGTATCCTTGAGCTGG
CGACTAACAAACGACACCAAAACATATCAGGCTGAAAAGGCTCGAGGACAACAAGCCTCAGGCATAGCATGCTATAT
GAAGGATAATCCAGGAGCAACTGAGGAAGATGCCATCAAGCACATATGTCGTGTTGTTGACCGGGCCTTGAAAGAAG
CAAGCTTTGAATATTTCAAACCATCCAATGATATCCCAATGGGTTGCAAGTCCTTTATTTTTAACCTTAGATTGTGT
GTCCAAATATTTTACAAGTTTATAGATGGGTACGGAATCGCCAATGAGGAGATTAAGGATTATATAAGAAAAGTTTA
TATTGATCCAATTCAAGTATGA

>CaGGPPS (mature)

ATGGCTACTACCAAAGAGGACGAAAGAATTGAAGCTGCTCAAACTGAAGAACCCTTCAACTTTAAGATCTACGTTAC
CGAAAAGGCCATCTCTGTTAACAAGGCTTTGGATGAAGCCATCATTGTCAAAGAACCACATGTTATCCATGAGGCCA
TGAGATATTCTTTGTTGGCTGGTGGTAAAAGGGTTAGACCAATGTTGTGTTTGGCTGCTTGTGAATTAGTTGGTGGT
AATCAAGAAAATGCTATGGCTGCTGCATGTGCCGTTGAAATGATTCATACAATGTCCTTGATCCACGATGATTTGCC
ATGTATGGATAACGATGACTTGAGAAGAGGTAAGCCAACTAACCATAAGATCTATGGCGAAGATGTTGCTGTTTTAG
CTGGTGATTCATTATTGGCTTTCGCCTTCGAACATATCGTTAATTCTACTGCTGGTGTTACCCCATCTAGAATAGTT
GGTGCTGTTGCTGAATTGGCTAAGTCTATTGGTACTGAAGGTTTGGTTGCTGGTCAAGTTGCTGATATTAAGTGTAC
TGGTAACGCCTCTGTTTCTTTGGAAACCTTGGAATTCATCCATGTTCATAAGACTGCTGCCTTGTTGGAATCTTCTG
TTGTTTTGGGTGCTATTTTAGGTGGTGGTACAAACGTTGAAGTCGAAAAGTTGAGAAGATTCGCTAGATGCATTGGT
TTGTTGTTCCAAGTTGTTGATGATATCCTGGATGTCACCAAGTCATCTGAAGAATTGGGTAAAACTGCTGGTAAGGA
TTTGGTTGTTGACAAGACTACTTACCCAAAGTTGTTGGGTCTAGAAAAGGCTAAAGAATTTGCTGCCGAATTGAACA
GAGAAGCCAAACAACAATTGGAAGGTTTCGATTCAAGAAAGGCTGCTCCATTGATTGCTTTGGCTGATTATATTGCC
TTGA

>SaGGPPS

ATGTCCTACTTCGATAACTACTTCAACGAGATCGTTAACTCCGTTAACGACATCATCAAGTCCTACATTTCTGGTGA
TGTTCCAAAGTTGTATGAAGCCTCTTACCATTTGTTTACCTCTGGTGGTAAAAGATTGAGGCCATTGATTTTGACCA
TCAGCTCTGATTTGTTTGGTGGTCAAAGGGAACGTGCTTATTATGCTGGTGCTGCTATTGAAGTCTTGCATACTTTT
ACTTTGGTTCACGATGACATCATGGACCAAGATAACATTAGAAGAGGTTTGCCAACCGTTCATGTTAAGTATGGTTT
GCCATTGGCTATTTTGGCTGGTGATTTGTTGCATGCTAAGGCTTTCCAATTATTGACTCAAGCCTTGAGAGGTTTAC
CATCCGAAACCATTATTAAGGCCTTCGATATTTTCACCAGGTCGATCATTATTATCTCCGAAGGTCAAGCTGTTGAC
ATGGAATTCGAAGATAGAATCGACATCAAAGAGCAAGAGTACTTGGACATGATCTCTAGAAAAACTGCTGCTTTGTT

CTCCGCCTCTTCTTCTATTGGTGCTTTGATTGCTGGTGCCAATGATAACGATGTTAGATTGATGTCTGACTTCGGTA
CAAACTTGGGTATTGCTTTCCAAATCGTTGATGACATTTTGGGTTTGACCGCTGACGAAAAAGAATTGGGTAAACCA
GTTTTCTCCGACATCAGAGAAGGTAAAAAGACCATCTTGGTTATCAAGACCTTGGAGTTGTGTAAAGAGGACGAAAA
GAAGATCGTTTTGAAGGCTTTGGGTAACAAGTCCGCTTCTAAAGAAGAATTGATGTCCTCCGCTGATATTATCAAGA
AATACTCTTTGGACTACGCCTACAACTTGGCTGAAAAGTACTACAAGAACGCCATCGATTCCTTGAATCAAGTCTCT
TCCAAATCCGATATTCCAGGTAAGGCTTTGAAATACTTGGCCGAATTCACTATCAGAAGGCGTAAATGA

>CrGGPPS (yellow highlight: truncation starts)

ATGCAAATGCAACAACAGAGAATGAGATTCTCCAGAGATGCTAGAAGAATTGGTGCTTCTAGAGTTCCATTGGCTAA
AGCTGCTCCAGGTAGAAAAGTTGTTGCTCAAGTTGCTACTGCTACCGCTGAAAAAGTTGATGTTAAGCAAGCTGGTT
CCTTCGATTTCAACACTTACATGGTTGATAGAGCCAAGTTGGTTAACAAGGCTATGGATGAAGCTGTTCCATTGAAA
TACCCAGAAACCTTGAACGAATCTATGAGGTATTCTTTGTTGGCTGGTGGTAAAAGGGTTAGACCAGCTTTGTGTTT
GGCTGCTTGTGAATTGGTTGGTGGTGATATTCATGCTGCTTTGCCAGCTGCATGTGCTATGGAAATGGTTCATACAA
TGTCCTTGATCCACGATGATTTGCCATCTATGGATAACGATGACTTCAGAAGAGGTAGACCAACTAACCATAAGGTT
TACGGTGAAGATATTGCTATTTTGGCCGGTGATGCTTTGTTGTCTTTTTCCTTTGAACATATTGCCAGAGCTACTAA
GGGTGTTCCAGCTGAAAGAGTTTTGAGAGTTATTATGGAATTGGGTAAAGCCGTTGGTCAAGATGGTTTGGTTGCTG
GTCAAGTTGTTGATATTCAATCCGAGGACAAAGAAGTTGGTTTGGACGTTTTGAAGTACATCCATGAACATAAGACT
GCAGCTTTGTTGGAAGCTAGTGTTGTTTGTGGTGCTATTTTAGGTGGTGCTGATGAATCTACCATTGAGAAGTTGAG
AAAGTACAGCTTGAACATTGGTTTGGCCTTCCAAGTTATCGATGACATTTTGGATGTTACCGCTACTACTGAACAGT
TGGGTAAAACTGCTGCTAAAGATTTGGCTGTTAACAAGACTACTTACCCAAAGTTGCTAGGTTTGGAAAAGTCTAAG
CAAGTTGCTGACGACTTGATCAAAGAAGCCATTCAACAATTGGATGGTTTCGATGCTGCAAAAGCTGCACCATTGGT
TGCTTTGGCTAAGTTTATTGGTTACAGGCAAAATTGA

>mERG20

ATGGCCTCCGAAAAAGAAATCAGAAGAGAAAGATTCTTGAACGTTTTCCCAAAGTTGGTTGAAGAATTGAACGCTTC
TTTGTTGGCATACGGTATGCCTAAAGAAGCTTGCGATTGGTACGCTCATTCTTTGAATTACAATACTCCAGGTGGTA
AGTTGAACAGAGGTTTGTCTGTTGTTGATACCTACGCCATTTTGTCTAACAAGACTGTAGAACAATTGGGTCAAGAA
GAATACGAAAAGGTTGCAATTTTGGGTTGGTGCATCGAATTATTGCAAGCTTACTGTTTGGTTGCCGATGATATGAT
GGATAAGTCCATTACTAGAAGAGGTCAACCATGTTGGTACAAAGTACCTGAAGTTGGTGAAATTGCTATCAACGATG
CTTTCATGTTGGAAGCTGCTATCTACAAGTTGTTGAAGTCCCATTTCAGAAACGAAAAGTATTACATCGACATCACC
GAATTATTCCACGAAGTTACTTTCCAAACCGAATTGGGTCAATTGATGGATTTGATTACTGCCCCAGAAGATAAGGT
TGACTTGTCTAAGTTCTCTTTGAAGAAGCACTCTTTCATCGTTACTTTCAAGACCGCTTACTACTCTTTCTATTTGC
CAGTTGCTTTGGCCATGTATGTTGCTGGTATTACTGACGAAAAGGATTTGAAGCAAGCCAGAGATGTTTTGATTCCA
TTGGGTGAATACTTCCAAATCCAAGATGATTACTTGGACTGTTTCGGTACTCCAGAACAAATTGGTAAGATCGGTAC
TGATATCCAAGACAACAAATGCTCCTGGGTTATTAACAAGGCTTTGGAATTGGCTTCTGCCGAACAAAGAAAAACTT
TGGACGAAAACTACGGTAAGAAGGATTCTGTTGCTGAAGCTAAGTGCAAGAAGATTTTCAACGACTTGAAGATCGAA
CAATTATACCACGAATACGAAGAATCCATTGCCAAAGATTTGAAGGCCAAGATTTCCCAAGTTGACGAATCTAGAGG
TTTCAAGGCTGATGTTTTGACTGCTTTTTTGAACAAGGTCTACAAGAGATCCAAGTAA
